# Nme_2_Cas9‐mediated therapeutic editing in inhibiting angiogenesis after wet age‐related macular degeneration onset

**DOI:** 10.1002/ctm2.1383

**Published:** 2023-08-20

**Authors:** Sihui Hu, Yuxi Chen, Dongchun Xie, Kan Xu, Yunzhao Fu, Wei Chi, Haiying Liu, Junjiu Huang

**Affiliations:** ^1^ MOE Key Laboratory of Gene Function and Regulation State Key Laboratory of Biocontrol School of Life Sciences Sun Yat‐sen University Guangzhou China; ^2^ The State Key Laboratory of Ophthalmology Zhongshan Ophthalmic Center Sun Yat‐sen University Guangzhou China; ^3^ Key Laboratory of Reproductive Medicine of Guangdong Province School of Life Sciences and the First Affiliated Hospital Sun Yat‐sen University Guangzhou China

**Keywords:** choroidal neovascularization, gene editing, gene therapy, Nme_2_Cas9, wet AMD

## Abstract

**Background:**

Age‐related macular degeneration (AMD), particularly wet AMD characterised by choroidal neovascularization (CNV), is a leading cause of vision loss in the elderly. The hypoxia‐inducible factor‐1α (HIF‐1α)/vascular endothelial growth factor (VEGF)/VEGF receptor 2 (VEGFR2) pathway contributes to CNV pathogenesis. Previous gene editing research indicated that disrupting these genes in retinal pigment epithelial cells could have a preventive effect on CNV progression. However, no studies have yet been conducted using gene editing to disrupt VEGF signalling after CNV induction for therapeutic validation, which is critical to the clinical application of wet AMD gene editing therapies.

**Method:**

Here, we employed the single‐adeno‐associated virus‐mediated Nme_2_Cas9 to disrupt key molecules in VEGF signalling, *Hif1α*, *Vegfa* and *Vegfr2* after inducing CNV and estimated their therapeutic effects.

**Results:**

We found that Nme_2_Cas9 made efficient editing in target genes up to 71.8% post 11 days in vivo. And only Nme_2_Cas9‐*Vegfa* treatment during the early stage of CNV development reduced the CNV lesion area by 49.5%, compared to the negative control, while Nme_2_Cas9‐*Hif1α* or Nme_2_Cas9‐*Vegfr2* treatment did not show therapeutic effect. Besides, no off‐target effects were observed in Nme_2_Cas9‐mediated gene editing in vivo.

**Conclusions:**

This study provides proof‐of‐concept possibility of employing Nme_2_Cas9 for potential anti‐angiogenesis therapy in wet AMD.

## Graphical abstract

1. Single AAV‐delivered Nme_2_Cas9 mediated efficient editing of wet AMD‐associated genes in vivo in a short time.
2. Nme_2_Cas9 targeting *Vegfa* gene significantly reduced the CNV lesion area by 49.5% after wet AMD onset with an early‐stage intervention, while Nme_2_Cas9 targeting *Hif1α* or *Vegfr2* did not show significant therapeutic effect.
3. No off‐target effects were observed in Nme_2_Cas9‐mediated gene editing in vivo.

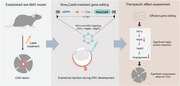

## 1 BACKGROUND

Clustered regularly interspaced short palindromic repeats (CRISPR) gene editing technology is expected to treat a variety of ophthalmic diseases by correcting or genetically modifying disease‐causing genes. The eye possesses several characteristics that make it the perfect target for gene therapy, including immune privilege, and ease of transduction by recombined virus vectors.^1^ Until now, ophthalmology is one of the leading fields in gene therapies with more than 90 clinical trials (CTs) as exampled by Luxturna and Edit‐101, the first in vivo human gene replacement therapy and gene editing therapy for the treatment of different types of Leber congenital amaurosis, respectively. Despite Luxturna's clinical success, there are still several limitations to gene replacement therapies, including the size of the target gene and the pathogenic alleles. Therefore, further attempts to employ novel gene regulatory and gene editing applications are crucial to targeting ocular diseases that have not been possible with the existing approaches, such as autosomal dominant retinitis pigmentosa, diabetic retinopathy and age‐related macular degeneration (AMD).

AMD is a progressive, degenerative disease of the retina that causes irreversible blindness in ageing people worldwide. AMD accounts for 8.7% of the worldwide population, and the projected number of people with the disease is around 196 million in 2020, expected to reach 288 million in 2040.^2^ AMD is classified into two categories: the atrophic (dry) form and the exudative neovascular (or wet) form, characterised by the presence of atrophic drusen and choroidal neovascularization (CNV), respectively.^3^ While atrophic AMD is common, neovascular AMD represents the most vision‐threatening form of the disease and accounts for almost 90% of blindness associated with AMD. Vascular endothelial growth factor (VEGF) is known as a major contributor to the pathogenesis of wet AMD, especially VEGFA. During the neoangiogenic phase of CNV, *VEGFA* is activated by a hypoxia‐inducible factor (HIF1α) under hypoxic conditions.^4^, ^5^ Then, VEGFA regulates angiogenesis and vascular permeability by activating two receptors on the surfaces of endothelial cells, VEGFR‐1 (Flt‐1) and VEGFR‐2, particularly VEGFR‐2, accelerating cell proliferation and migration and so promoting the development of new blood vessels.^6^

Current treatments for wet AMD with anti‐VEGF drugs include bevacizumab,^7^ ranibizumab,^8^ aflibercept^9^ and brolucizumab.^10^ However, there exist some concerns of adverse effects with long‐term repeated administration, such as chorioretinal atrophy.^11^, ^12^ Gene therapies for wet AMD were born to gain more sustained treatments and overcome the burden of repeated injections. Most current gene therapy strategies employ a biofactory approach of transducing retinal cells with viral vectors, such as lentiviruses (LVs) and adeno‐associated viruses (AAVs) to produce VEGF antagonists. To date, one LV‐based gene therapy CT^13^ and 11 AAV‐based gene therapy CTs (https://clinicaltrials.gov/ct2/) have been developed to treat wet AMD. However, these strategies are in their early stages and have not been evaluated for long‐term efficacy.

CRISPR/CRISPR‐associated protein 9 (Cas9) is currently a fundamental tool for both basic and translational research. Using CRISPR/Cas9, gene expression products can be modulated by producing mutations in the gene loci associated with angiogenesis, such as *Vegfa* and *Hif1α* to dramatically suppress angiogenesis permanently, with viral vectors, such as AAVs, to diseased tissues to treat different ocular diseases.^14^ Some efforts have been made by using CRISPR/Cas9 to target these proangiogenic signals in preclinical models of wet AMD and show significant effects of prophylactic treatment in laser‐induced CNV. Kim and colleagues used the smaller Cas9 protein derived from *Campylobacter jejuni* (CjCas9) and the RNA‐guide nuclease, CRISPR from *Prevoltella* and *Francisella* (Cpf1) to disrupt *Vegfa* and *Hif1α* gene before laser‐induced CNV and found that it significantly mitigated mouse CNV progress, demonstrating that suppressing the angiogenesis pathway is effective for the treatment of wet AMD.^15^, ^16^ Huang et al. using *Streptococcus pyogenes* Cas9 (SpCas9) to edit the VEGF receptor 2 (*Vegfr2*) locus also attenuated the disease phenotype of wet AMD.^17^ However, there have been no studies yet using gene editing to intervene in the VEGF signal after CNV induction for therapeutic validation, and it is unknown whether using gene editing therapy to block the VEGF pathway after AMD onset is effective.

Here, we used a single‐AAV delivery Nme_2_Cas9 to screen effective‐editing targets of three key molecules in VEGF signalling, *Hif1α, Vegfa and Vegfr2* and validated their editing efficiency in vivo. Subsequently, we disrupted each of the three molecules at different times after laser‐induced CNV and evaluated the CNV lesion to study the effects of blocking VEGF signalling after wet AMD onset. Besides, we assessed the potential off‐target effect of Nme_2_Cas9 to investigate the safety of Nme_2_Cas9 in vivo. This research provides a basis for the clinical translational application of gene editing‐based gene modulation therapies for wet AMD.

## 2 MATERIALS AND METHODS

### 2.1 gRNA and AAV vector design

CRISPR single‐guide RNAs (sgRNAs) for *Neisseria meningitidis* Cas9 (Nme_2_Cas9) were computationally designed by using Cas‐Designer (http://www.rgenome.net/cas‐designer/) to determine sgRNAs with high on‐target binding specificity. Four sgRNAs targeting important functional domains of proteins for each gene were selected. Target sequences were cloned into the all‐in‐one AAV plasmid expressing Nme_2_Cas9 (Addgene plasmid# #119924) via SapI (New England Biolabs) restriction enzyme sites upstream of the scaffold sequence of the U6‐driven sgRNA cassette.^18^ Sequences of sgRNAs are listed in Supplemental Table S1.

### 2.2 Cell culture and transfection

Mouse E14 cells and human HEK293T were purchased from ATCC. Mouse E14 cells were cultured in 0.2% gelatine‐treated dish with medium containing Knockout Dulbecco's modified eagle medium (DMEM) (Gibco) and 20% fetal bovine serum (FBS) (Hyclone, Thermo), 1% Penicillin–streptomycin (Gibco), 1% L‐glutamine (Gibco), 1% non‐essential amino acid (Gibco), 0.1 mM β‐mercaptoethanol (Sigma‐Aldrich) and incubated at 37°C and 5% atmospheric CO_2_ and 100% relative humidity. HEK293T cells were cultured in DMEM (Corning) and 10% FBS (Lonsera). Polyethylenimine, linear (PEI) was used for transfection in HEK293T, while transfection in E14 cells was performed using LipoFectMax 3000 (ABP biosciences, FP319M). Cells were collected 3 days after transfection for further analysis.

### 2.3 T7E1 assay

Genomic DNA was extracted from infected cells and tissues utilising a commercially available kit (AxyPrep Blood Genomic DNA Miniprep Kit, Axygen) according to the manufacturer's instructions. Subsequently, the concentration of the genomic DNA was measured using a spectrophotometer (NanoDrop One, Thermo). The specific gene target regions were amplified through polymerase chain reaction (PCR) using high‐fidelity DNA polymerase (KOD, Toyobo) and primers designed to flank the target sites (Supplemental Table S2). The 200 ng purified PCR products were denatured, and the annealing intensities were measured with a thermal cycler using the following program settings: 95°C for 5 min, −2°C/s from 95 to 85°C, −0.1°C/s from 85 to 25°C. T7 endonuclease I (New England Biolabs) was incubated with annealed PCR products for 45 min at 37°C before being evaluated by agarose gel electrophoresis. ImageJ (http://imagej.nih.gov/ij/) was used to assess band intensities, and the proportion of indel was measured as previously described.

### 2.4 AAV preparation

AAV package and quantification were handled following Addgene's protocols. Briefly, HEK293T cells were cultured in a 15‐cm dish until 75% confluency and transfected with 8‐μg of inverted terminal repeat (ITR) containing transfer plasmid, 13‐μg helper plasmid, and 14‐μg AAV2 Rep/AAV8 Cap AAV packaging vectors mixed with PEI. Ninety‐six hours after transfection, the supernatant was collected and processed with polyethylene glycol (PEG) 8000 (Sigma) for virus precipitation. Sonication and benzonase treatment were used to collect and lyse the cells. Then, for further purification of AAVs, an iodixanol gradient (Sigma) was used for ultracentrifugation. Fractions containing AAVs were collected and dialysis against 0.001% Pluronic F68 in phosphate buffer saline (PBS). Finally, titers of the virus were estimated using primers listed on Addgene (ITR FP: GGAACCCCTAGTGATGGAGTT; ITR FP: CGGCCTCAGTGAGCGA) by quantitative PCR. The purified virus was then aliquoted and stored at −80°C.

### 2.5 Animals

The 6–8 weeks old C57BL/6j male mice were used in this research. The mice were fed in a pathogen‐free animal facility under conventional circumstances (22 ± 1°C) with a 14/10 h light–dark cycle at Sun Yat‐sen University. All animal experiments were approved by the Institutional Animal Care and Use Committee of Sun Yat‐sen University, P. R. China.

### 2.6 Laser‐induced CNV model

An intraperitoneal dose of pentobarbital (50 mg/kg body weight) was used to anaesthetise the mice. An eye drop containing phenylephrine (0.5%) and tropicamide (0.5%) was used to dilate the pupils. To observe the entire eye fundus, a drop of ophthalmic viscoelastic solution was added to the corneal surface. An indirect headset delivery system (Iridex, SLX) and a laser system (IRIS Medical) were used to carry out laser photocoagulation; 532‐nm wavelength, 75‐m spot size, 250‐mW power, and 100‐ms exposure time were the laser parameters. Four laser burns were generated around the optic disc. Only burns that produced a bubble without vitreous hemorrhage were included in the study.

### 2.7 Subretinal injection in mice

The subretinal injection was performed as previously described.^19^ Briefly, 1‐μL AAV8 (9 × 10^9^ VG Nme_2_Cas9 with sgRNA + 1 × 10^9^ VG green fluorescent protein (GFP)) was injected into the subretinal region in the 40 s using a 33G blunt‐end needle (Hamilton, 7803–05) and a Hamilton syringe (1701RN No Needle 7653‐01). Then, tobramycin (Alcon) was used for preventing dryness and post‐surgery infection.

### 2.8 Isolation of retinal pigment epithelial (RPE) sheets and genomic DNA extraction

Following the previously described process, the entire RPE cell sheet post injection was harvested.^20^ Then, the collected RPE cells were incubated with lysis buffer (QuickExtract Solution, Lucigen) for 20 min at 65°C, 2 min at 95°C and then stored at −20°C. Targeted Sanger sequencing and deep sequencing were performed on the genomic DNA.

### 2.9 Immunofluorescence staining and imaging of choroid flat mount

Further research was conducted on subjects in which the AAV‐GFP overlapped the laser‐burn location. The eyeballs were fixed in 1% paraformaldehyde (PFA) for 1 h at room temperature post injection. RPE complexes were applied for overnight staining at 4°C with isolectin‐B4 (Thermo, Catalog No. I21413, 1:50), Hif1a (Santacruz, Catalog No. sc10790, 1:100), Ca9 (Abcam, Catalog No. ab243660, 1:100) or Cd11b (Abcam, Catalog No. ab8878, 1:200). The RPE complexes were flat‐mounted and magnified 20× using a fluorescent microscope (Observer 5, Zeiss). The CNV area was assessed blindly using ImageJ program.

### 2.10 Mouse VEGFA enzyme‐linked immunosorbent assay (ELISA)

A total of 8–12 eyes were used as samples for each group. And four laser burns were induced in each eye after Nme_2_Cas9‐*Vegfa*, or GFP AAVs (1‐μL) were injected into the subretinal space. Whole RPE complexes were extracted from the retina at 11 days after injection and stored for further investigation. Cells were lysed in 120‐μL radio immunoprecipitation assay (RIPA) buffer, and VEGFA levels were assessed using a mouse VEGF Quantikine ELISA Kit (MMV00, R&D systems) as directed by the manufacturer.

### 2.11 Electroretinography (ERG)

The mice were dark‐adapted for 16 hours before recording ERG. Pentobarbital (50 mg/kg body weight) was used to anaesthetise mice. And 1% tropicamide was used to dilate their pupils. The RetiMINER‐C (IRC) was used to record ERG in accordance with the manufacturer's instructions. To keep the cornea moist, a drop of saline was applied. For scotopic ERG, mice were dark‐adapted overnight and stimulated with flashes of steadily increasing light intensity (0.001, 0.01, 0.1, 1, 3 and 10 cd*s/m^2^). For photopic ERG, mice were light‐adapted for 10 min and stimulated with flashes (1, 3 and 10 cd*s/m^2^) while white background light (30 cd*s/m^2^) was presented concomitantly. The band‐pass filter was set between 0.3 and 300 Hz, and responses were averaged from three single flashes. All data were analysed via RetiMINER4.0. The a‐wave amplitude was measured from the baseline to the lowest negative‐going voltage, whilst peak b‐wave amplitudes were measured from the a‐wave trough to the highest positive b‐wave peak.

### 2.12 In silico prediction of off‐targets

Cas‐OFFinder was used for off‐target identification in the hg38 reference genome. The protospacer sequence was adjusted to simulate DNA and RNA bulges by either removing single bases or adding gaps (indicated by N in the sequence). Supplementary Table S3 contains a list of potential off‐target sites.

### 2.13 Targeted deep sequencing analysis

The target/off‐target sites were amplified with barcode‐containing primers and a KOD PCR kit (TOYOBO) to generate a deep sequencing library. PCR products were then sequenced paired‐end 150 Hiseq 2000342 (Illumina). Samples with reads over 10 000 were calculated. Indel frequency was analysed by CRISPResso2.

### 2.14 Statistics

Utilising GraphPad Prism 8.0.2, data were examined. In all of the experiments (*n* ≥ 3), data are provided as mean ± s.e.m. The *p*‐values (95% confidence interval) were determined using one‐way ANOVA and the Dunnett's test. The figure labels described the specific statistical method used as well as the replicates. The asterisks indicate statistical significance; unless otherwise specified, *p < .05, **p < .01, ***p < .001; ns, not significant.

## 3 RESULTS

### 3.1 Nme_2_Cas9‐mediated efficient editing of wet AMD‐associated genes in mouse cells

**FIGURE 1:**
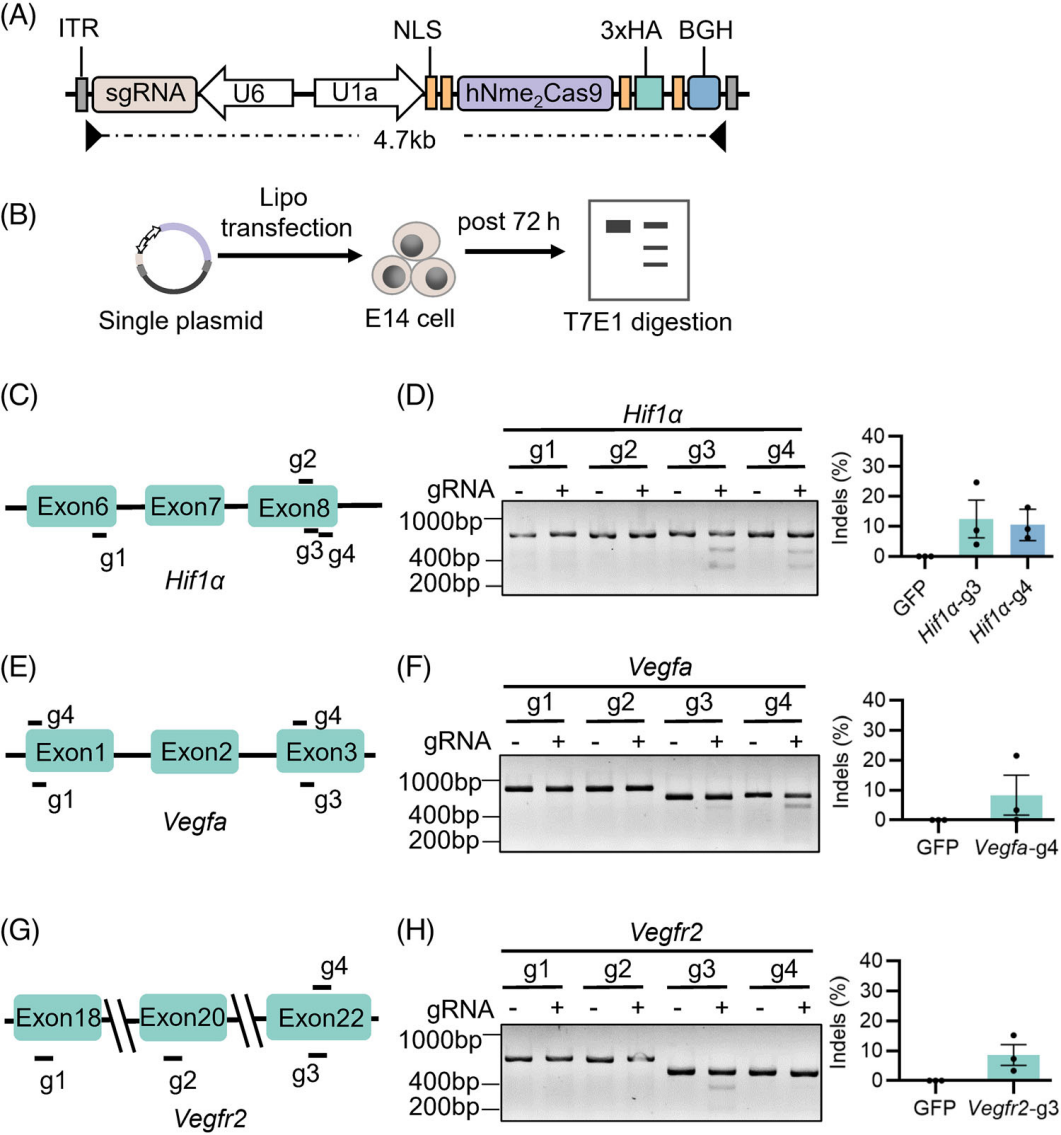
Screening efficient sgRNAs targeting wet age‐related macular degeneration (AMD)‐associated genes with Nme_2_Cas9 in mouse cells. (A) Schematic illustration of the all‐in‐one adeno‐associated virus (AAV) vector expressing Nme_2_Cas9 and the sgRNA. ITR, inverted terminal repeat; BGH, bovine growth hormone poly(A) site; HA, epitope tag; NLS, nuclear localisation sequence; h, human‐codon‐optimised. (B) Editing efficiency detection of sgRNAs and Nme_2_Cas9 in mouse E14 cells with T7 endonuclease 1 (T7E1) assay. (C–H) Schematic illustration of *Hif1α* (C), *Vegfa* (E) and *Vegfr2* (G) genes and four targeted sites of Nme_2_Cas9 sgRNA candidates on the genes. Cleavage efficiencies (%indel) of sgRNAs for *Hif1α* (D), *Vegfa* (F) and *Vegfr2* (H) genes were tested by T7E1 assay, displayed as mean ± s.e.m. Cells transfected with GFP served as a negative control. Representative results are shown from three biological repeats. +, treated with Nme_2_Cas9 and sgRNA; –, treated with GFP.

First, we selected three genes (*Hif1α*, *Vegfa* and *Vegfr2*) as the editing targets that could effectively inhibit the expression of angiogenesis‐related genes. Here, we used a small Cas9 ortholog derived from *Neisseria meningitidis* (Nme_2_Cas9) that could be packaged with sgRNA into a single AAV,^21^ and designed four sgRNAs targeting the relatively conservative functional domains of *Hif1α*, *Vegfa* and *Vegfr2* genes, to effectively silence wet AMD‐associate genes (Figure 1A). After 72 h, the mouse embryonic stem cells (E14) were harvested for genomic DNA extraction, target sequences PCR and T7 Endonuclease 1 (T7E1) digestion (Figure 1B). The PAS_3 conserved domain in Hif1α has been shown to bind ligands and serve as sensors for light and oxygen in signal transduction.^22^ We observed that two sgRNAs (*Hif1α*‐g3 and g4) targeting this domain were effective, and *Hif1α*‐g3 performed better with 12.49% indels (Figure 1C,D). The *Vegfa*‐g4 sgRNA targeting platelet‐derived and vascular endothelial growth factors (PDGF) domain showed high efficiency for the *Vegfa* gene with 8.35% indels (Figure 1E,F). Additionally, The *Vegfr2*‐g3 sgRNA targeting the catalytic domain of the protein tyrosine kinase proved effective for the *Vegfr2* gene with 8.62% indels (Figure 1G,H). Based on the results above, we decided to use *Hif1α*‐g3, *Vegfa*‐g4 and *Vegfr2*‐g3 sgRNAs to continue our research.

### 3.2 Single AAV‐delivered Nme_2_Cas9‐mediated efficient editing in mouse RPE cells

**FIGURE 2:**
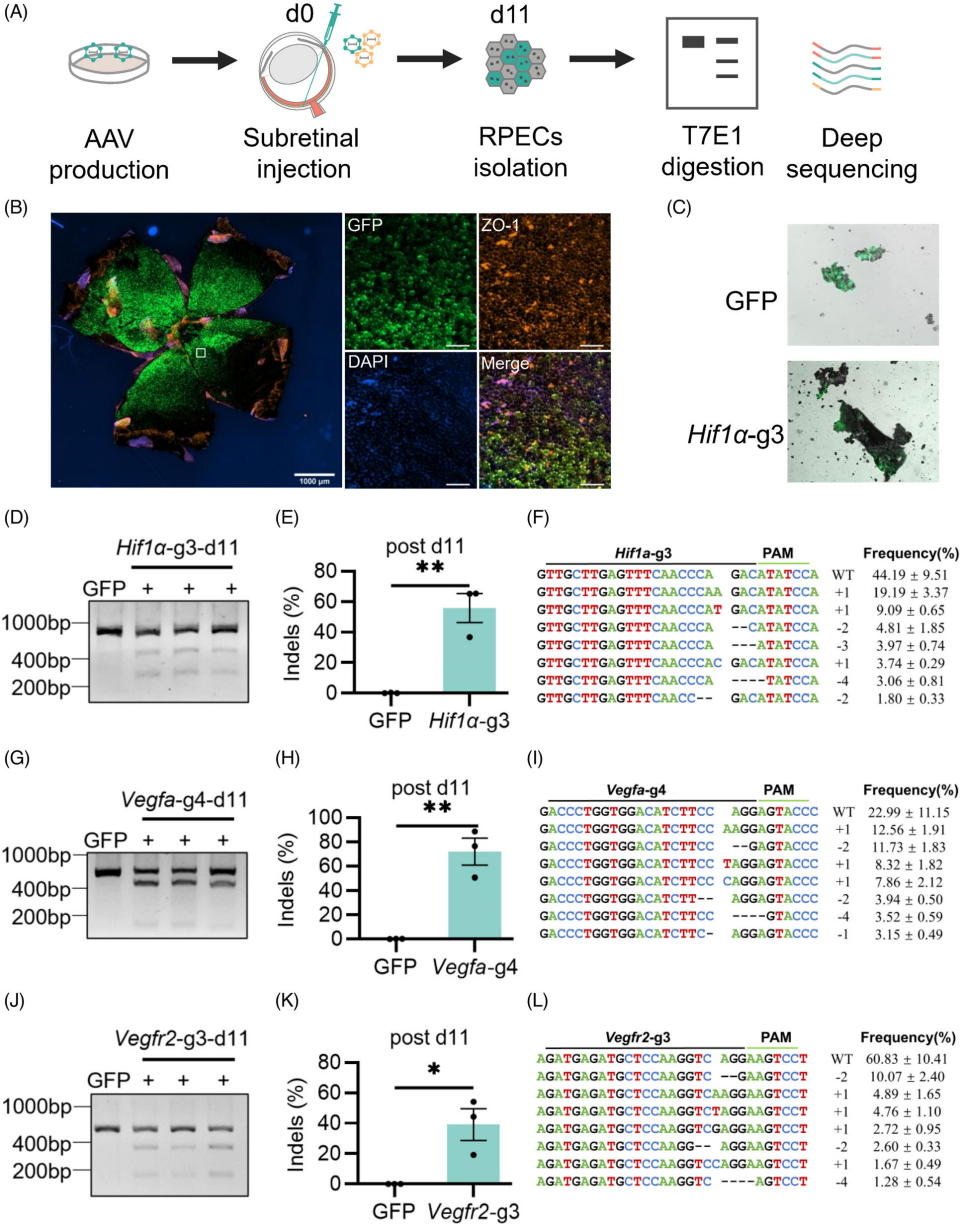
Detecting gene editing efficiency of single AAV‐mediated Nme_2_Cas9 in mouse retinal pigment epithelial (RPE) cells. (A) AAV packaging and editing efficiency analysis of single AAV‐delivered Nme_2_Cas9 in mice. 1 × 10^10^ vg AAV8 mix was injected into mouse retina. Mouse RPE cells’ genomic DNA was extracted 11 days post injection, then editing efficiency was qualified. d, day. (B) Immunofluorescence staining of an RPE flat mount with AAV8‐GFP. Tight junction protein (ZO‐1) staining showed RPE cells’ morphology. Scale bar, 100 μm. (C) RPE cell isolation. (D–L) Cutting efficiency results detected by T7E1 assay at *Hif1α*‐g3 (D), *Vegfa*‐g4 (G) and *Vegfr2*‐g3 (J). +, represented an individual sample. Deep sequencing results at the *Hif1α*‐g3 site(E), *Vegfa*‐g4 site(H) and *Vegfr2*‐g3 site (K). GFP served as a negative control. Error bars indicate s.e.m. (*n* = 3). Unpaired Student's *t*‐tests, *p < .05, **p < .01. Top eight indel profiles of *Hif1α*‐g3 (F), *Vegfa*‐g4 (I) and *Vegfr2*‐g3 (L). +, insertion; –, deletion.

Next, we investigated the editing efficiency of the single AAV‐mediated Nme_2_Cas9 in vivo. We packaged Nme_2_Cas9 and sgRNA into a recombined AAV8 capsid to target *Hif1α*, *Vegfa* and *Vegfr2* genes in the mouse retina. To indicate the infection area, AAV8‐GFP was mixed with AAV8‐Nme_2_Cas9‐sgRNA. Then, AAVs (9 × 10^9^ vg Nme_2_Cas9 with sgRNA + 1 × 10^9^ vg GFP) were delivered to the mouse by subretinal injection. RPE cells of the whole flat mount, which were the primary target cells for the treatment of wet AMD, were isolated, and the editing efficiency was detected by T7E1 assay and next‐generation sequencing 11 days post injection (Figure 2A–C). Nme_2_Cas9 performed effective editing on *Hif1α* in all treated mice at Day 11 (Figure 2D), and the average indel efficiency was 55.64% ± 9.51% as revealed by targeted deep sequencing (Figure 2E), and more than 95% of the indels were frameshift mutations (Figure 2F). Similar results were observed on *Vegfa*‐g4 and *Vegfr2*‐g3 with average indel efficiencies of 71.80% ± 11.15% and 39.11% ± 10.41% respectively, both of which with more than 95% frameshift mutations (Figure 2G–L). In addition, the indel efficiency of *Vegfr2*‐g3 increased to 62.61% ± 12.07% with similar mutations at Day 15 (Figure S1A–D). These data showed that endogenous genomic sites in adult mouse retinas could be efficiently edited by Nme_2_Cas9 delivered by AAV rapidly.

### 3.3 Effective therapeutic editing of *Vegfa* suppressed laser‐induced CNV by single AAV‐delivered Nme_2_Cas9

In studies of the exudative form of AMD, the laser‐induced CNV mouse model has been employed extensively.^23^, ^24^ To estimate the therapeutic effects of CNV inhibition by AAV‐mediated Nme_2_Cas9, we generated a CNV mouse model by laser induction and traced its development (Figure S2A). By comparing the CNV lesion area labelled by isolectin‐B4 of the RPE flat mount at different times, we found that the CNV developed and receded fast, reaching a peak around Days 7 and 10 (Figure S2B) corresponding to previous research.^25^

**FIGURE 3:**
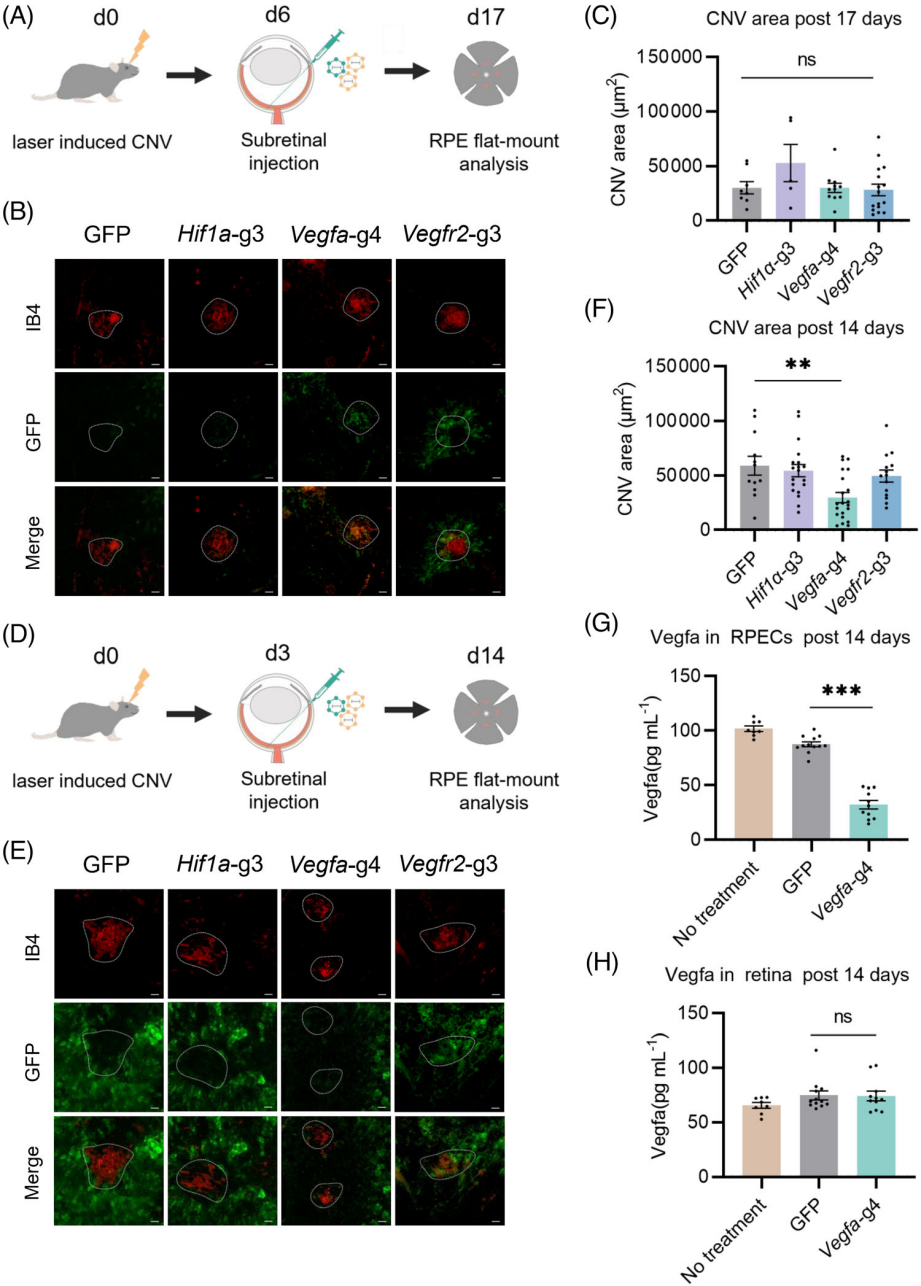
Therapeutic editing of *Vegfa* suppressed laser‐induced choroidal neovascularization (CNV) by single AAV‐delivered Nme_2_Cas9. (A) Schematic illustration of single AAV‐delivered Nme_2_Cas9 treatment for laser‐induced CNV in mice. At 6 days post laser treatment, 1 × 10^10^ vg AAV8 (9 × 10^9^ vg Nme_2_Cas9 with gRNA+ 1 × 10^9^ vg GFP) was injected into the mouse retina. Eleven days after subretinal injection, the CNV area was analysed. (B) Representative images of laser‐induced CNV stained with anti‐IB4 antibody (red) in the mouse eyes injected with AAV8‐GFP or AAV8‐Nme_2_Cas9 (+ 1 × 10^9^ vg AAV8‐GFP) targeted to *Hif1α*, *Vegfa* or *Vegfr2* (green). Scale bar, 50 μm. (C) The CNV area. Error bars indicate s.e.m. (*n* = 5–16). One‐way ANOVA with the Dunnett's test, *p < .05; **p < .01; ***p < .001; ns, not significant. (D) Schematic illustration of single AAV‐delivered Nme_2_Cas9 treatment for laser‐induced CNV in mice at an early stage. At 3 days post laser treatment, 9 × 10^9^ vg AAV8‐Nme_2_Cas9 with 1 × 10^9^ vg GFP was injected into the mouse retina. Then, 11 days after subretinal injection, the CNV area was analysed. (E) Representative images of laser‐induced CNV stained with anti‐IB4 antibody (red) in the mouse eye injected with AAV8‐GFP or AAV8‐Nme_2_Cas9 (+ 1 × 10^9^ vg AAV8‐GFP) targeted to *Hif1α*, Vegfa or *Vegfr2* (green). Scale bar, 50 μm. (F) The CNV area. Error bars indicate s.e.m. (*n* = 12–21). One‐way ANOVA with the Dunnett's test, *p < .05, ns, not significant. (G) Vegfa levels were measured by ELISA in the RPE cells. Error bars indicate s.e.m. (*n* = 8–12). One‐way ANOVA with the Dunnett's test, *p < .05; **p < .01; ***p < .001; ns, not significant. (H) Vegfa levels were measured by ELISA in the mouse retina. Error bars indicate s.e.m. (*n* = 8–12). One‐way ANOVA with the Dunnett's test, *p < .05; **p < .01; ***p < .001; ns, not significant.

Hence, we treated the laser‐induced CNV by subretinal injecting Nme_2_Cas9 on Day 6 with sgRNAs targeting *Hif1α*, *Vegfa* and *Vegfr2*. Then, the flat‐mounted choroids were isolated 11 days after the AAV injection (Figure 3A). No significant difference was observed in these groups, compared to the negative control, while the CNV area with Nme_2_Cas9*‐Vegfa* treatment showed a decreasing trend (Figure 3B, C). Since the three factors are rapidly upregulated during the early stages of the development of CNV,^5^ therapeutic interventions via AAV on Day 6 might be too late to suppress angiogenesis.

We assumed that earlier intervention with AAV would improve CNV suppression. Therefore, we injected AAV 3 days post laser treatment, and analysed the eyes for Nme_2_Cas9‐mediated gene editing effects 11 days after AAV injection (Figure 3D). Only Nme_2_Cas9‐*Vegfa* (59056 μm^2^) treatment significantly reduced the CNV area size by 49.52% ± 29.59%, compared with the negative control (29810 μm^2^), while no significant difference was observed in Nme_2_Cas9‐*Hif1α* or Nme_2_Cas9‐*Vegfr2* treatment (Figure 3E, F). Since the Vegfa protein produced and secreted by RPE cells is thought to be a key factor in inducing CNV,^26^, ^27^ we used the ELISA to compare Vegfa protein expression in RPE cells with or without Nme_2_Cas9‐*Vegfa* treatment. Vegfa protein expression level was reduced by 63.20% in RPE cells treated with Nme*_2_* Cas9‐*Vegfa* (32.24 pg/mL) versus those treated with GFP (87.51 pg/mL; Figure 3G). Besides, Vegfa protein expression levels in retinal tissues treated with Nme_2_Cas9‐*Vegfa* were nearly identical to those in retinal tissues treated with GFP (Figure 3H). The oxidative stress, inflammatory cells and pathways play critical roles in the pathogenies of CNV and could proceed upregulation of VEGF signalling. So, we also evaluated the inflammatory and oxidative responses following injecting Nme_2_Cas9‐*Vegfa*. The quantitative real‐time PCR and immunofluorescence of RPE flat mounts both showed that injecting AAV‐Nme_2_Cas9‐*Vegfa* could downregulate inflammatory and oxidative responses significantly (Figures S3 and S4). Besides, we evaluated the potential toxicity of Nme_2_Cas9‐*Vegfa* to retinal function by full‐field electroretinography (ERG) in mice at 11 days after the subretinal injection of Nme_2_Cas9‐*Vegfa*. We observed no significant decrease in the scotopic response in these mice, compared to the untreated mice (Figure S5). Therefore, AAV8‐delivered Nme_2_Cas9 mainly disrupted the *Vegfa* gene in RPE cells, not other retina cells, and suppressed CNV after wet AMD onset.

### 3.4 Safety assessment of Nme_2_Cas9‐mediated gene editing in RPE cells

**FIGURE 4:**
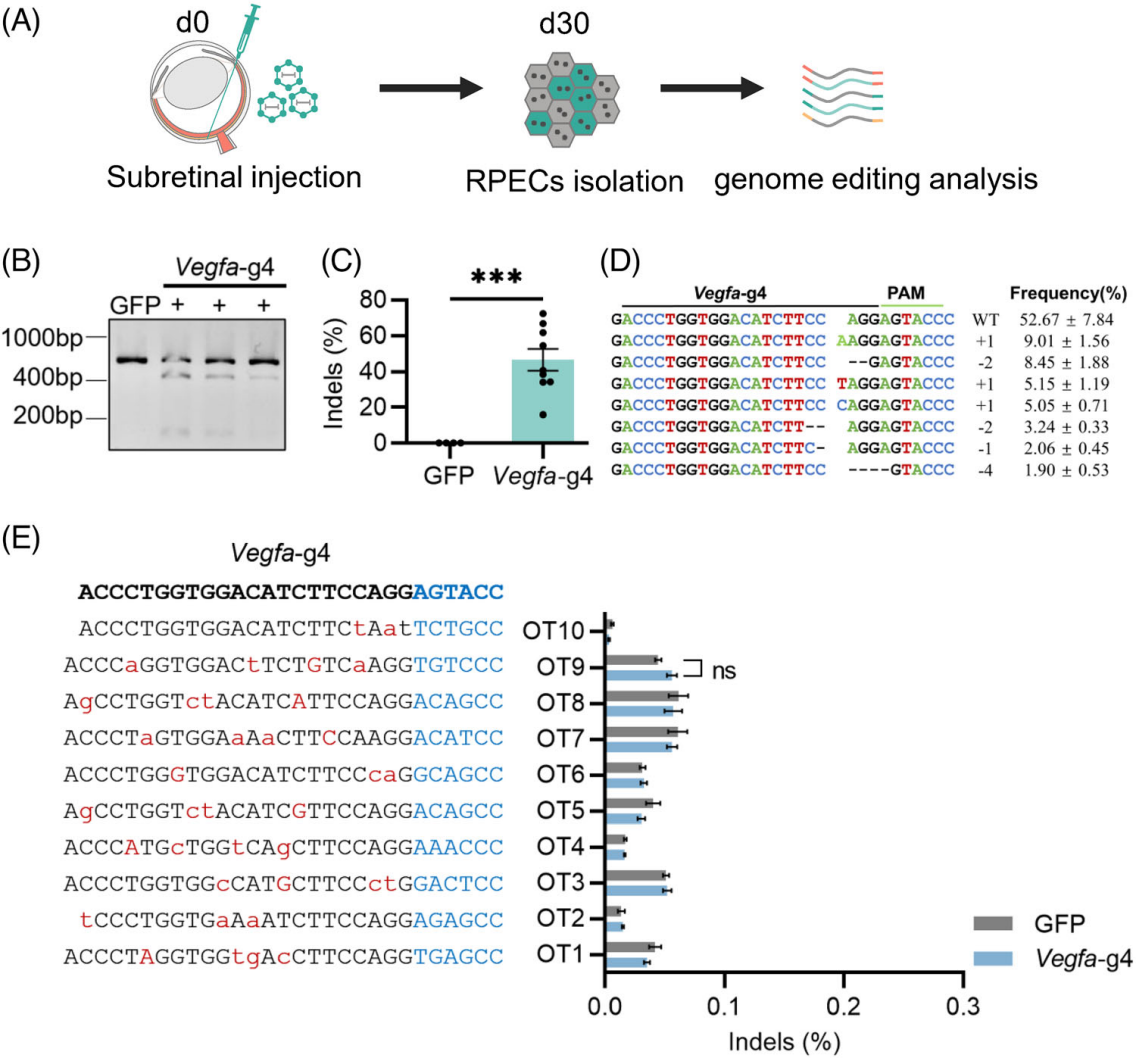
Safety assessment of single AAV‐mediated Nme_2_Cas9 targeting *Vegfa* gene in mouse RPE cells. (A) At 30 days post AAV8 mix injection, the mouse RPE cells’ genomic DNA was extracted for genome editing analysis including on‐target editing and potential off‐target editing efficiency. (B) T7E1 assay showed the cutting efficiency of AAV8‐Nme_2_Cas9 with *Vegfa*‐g4 in RPE cells. GFP served as a negative control. +, represented an individual sample. (C) Indel frequencies at the *Vegfa*‐g4 site were measured through targeted deep sequencing. Error bars indicate s.e.m. (GFP, *n* = 4; *Vegfa*‐g4, *n* = 9). Unpaired Student's *t*‐tests, *** < .001. (D) The indel profile of *Vegfa*‐g4 in mouse RPE cells. The frequencies of the top eight were analysed. +, insertion; –, deletion. (E) The indel ratio of predicted off‐target sites of *Vegfa*‐g4 in mouse RPE cells (GFP, *n* = 4; *Vegfa*‐g4, *n* = 9) was revealed by targeted deep sequencing. ns, not significant.

To assess the potential off‐target effects of Nme_2_Cas9‐mediated gene therapy in vivo, we injected AAV8‐Nme_2_Cas9 targeting *Vegfa* into the adult mouse retina. After 30 days, the whole RPE cells were collected for genomic DNA extraction and PCR amplification of target sites and potential off‐target sites (Figure 4A). First, we confirmed the editing effect of Nme_2_Cas9 aiming target *Vegfa*, which was similar to the previous detection in vivo with 46.45% ± 9.44% indels, and most of the indels were frameshift mutations (Figure 4B–D). As expected, the single AAV‐mediated Nme_2_Cas9 showed prominent gene editing efficiency for target endogenous sites in mouse RPE cells. Next, we estimated the off‐target efficacy of Nme_2_Cas9 in vivo. We used the Cas‐OFFinder,^28^ which is fast and relatively reliable. We selected 10 potential off‐target sites for *Vegfa*‐g4 with no more than three mismatches and no more than two DNA bulge sizes. Then, the sites were amplified for deep sequencing. Through analysing the potential off‐target sites with CRISPResso2,^29^ we did not observe off‐target effects for the gRNA targeting *Vegfa* (Figure 4E). Meanwhile, the on‐target and off‐target editing effects of *Hif1α*‐g3, *Vegfr2*‐g3 30 days after AAV injection were estimated. The on‐target editing efficiencies of Nme_2_Cas9 targeting *Hif1α* and *Vegfr2* were 42.29% ± 10.52% and 24.59% ± 5.98% indels, respectively (Figure S6A–F). And no off‐target effects for the gRNAs targeting *Hif1α* and *Vegfr2* were detected either (Figure S6G,H). Therefore, Nme_2_Cas9‐mediated gene editing in vivo showed no off‐target edits in most matched loci.

## 4 DISCUSSION

In this proof‐of‐concept study, we present data that a single AAV‐delivered Nme_2_Cas9 efficiently edited the *Hif1α*, *Vegfa* and *Vegfr2* genes with up to 71.80% editing efficiency in vivo in a short time without off‐target edits (Figures 2 and 4). We evaluated the effects of targeting different molecules in VEGF signalling by gene editing tool after wet‐AMD onset and found that only an early intervention of *Vegfa* at the neoangiogenic phase of CNV could effectively suppress the angiogenesis in a short time with a reduction of VEGF protein by 63.2% and the CNV area by 49.5% (Figure 3), while intervention of *Hif1α* and *Vegfr2* did not show significant therapeutic effect. Besides, The AAV8‐Nme_2_Cas9‐*Vegfa* treatment downregulated inflammation and oxidative responses significantly. These have demonstrated that reducing VEGFA after CNV induction showed distinct and effective intervention of angiogenesis pathway for treating wet AMD. The ERG results reflected no retinal dysfunction after subretinal injection of Nme_2_Cas9‐*Vegfa*, further proving that Nme_2_Cas9‐mediated gene editing therapy for wet‐AMD was safe. The efficacy and safety assessment of the gene editing therapy in a more accurate application scenario would provide a suitable reference for the clinical translation of wet‐AMD gene therapy researches. In addition, our research also proved that Nme_2_Cas9 as an effective tool for gene editing therapy in vivo.

Previous studies have shown that disruption of HIF1A before induction of CNV alleviated the disease development.^15^, ^16^ However, in our study, we found that intervention of HIF1A may no longer be sufficient to inhibit angiogenesis when VEGFA transcription has been activated and CNV has formed. VEGF‐A regulates angiogenesis and vascular permeability by activating 2 receptors, VEGFR‐1 (Flt‐1) and VEGFR‐2.^6^ Our data showed that only intervention through targeting the *Vegfa* gene at the neoangiogenic phase of CNV could promote the cure of wet AMD, while disrupting the *Vegfr2* gene slightly but not significantly reducing CNV lesion area, indicating that targeted disruption of *Vegfr2* was not as efficient as *Vegfa*. But it was uncertain whether the destruction of the *Vegfr2* gene had a long‐term effect on suppressing CNV due to the short observation period of the laser‐induced CNV model, which was only more than 10 days. Considering the low specificity of AAV8 targeting vascular epithelial cells, it is necessary to change AAV serotypes and assess the editing effectiveness of targeting *Vegfr2* and the therapeutic effect of reducing Vegfr2 expression in vascular epithelial cells after wet AMD onset in a subsequent study. These results suggested that there was a significant difference in the efficacy of different targets as therapeutic applications, and thus the selection of appropriate targets during disease progression requires further investigation.

For most patients with wet AMD, long‐term VEGF inhibition may be necessary.^27^, ^30^ However, VEGF is an important neurovascular trophic factor that is required for neuronal and endothelial cell survival and function, in addition to having angiogenic activity.^31^, ^32^ Long‐lasting but non‐specific VEGF antagonism may be detrimental to the health of normal cells that depend on its trophic activity. Some adverse effects have occasionally been observed in patients with sustained and frequent intravitreal injections of VEGF‐neutralising antibodies in clinical studies.^12^, ^33^, ^34^ It has also been reported that persistently suppressing VEGF expression in whole RPE cells could cause choroidal capillary shrinkage, leading to progressive degeneration marked by abnormalities in the RPE and brunch membrane, eventually resulting in RPE loss, significant choroidal remodelling and thickness reduction of retinal tissues.^26^, ^35^ Previously, Kim et al. explored the prophylactic efficacy of gene editing therapies for wet AMD by pre‐disrupting *Vegfa* with AAV9‐delivered CjCas9 and evaluated the long‐term safety concerns of this approach. It is worth noting that this strategy made 20% ∼ 30% indels in RPE cells and ∼40% indels in retina, and the retinal tissue was thinner at 14 months after injection.^36^ Upregulated VEGFA expression in RPE cells is critical in promoting the development of CNV.^27^, ^37^ Therefore, precise modification of *Vegfa* in RPE cells near CNV lesion to inhibit angiogenesis may be more beneficial for patients with wet AMD. In our study, we performed a therapeutic intervention after wet AMD onset and observed that reducing VEGFA expression by locally delivering a gene‐editing system in the early stages of CNV development is effective in treating CNV in a short time. And the *Vegfa* gene was a more direct and efficient target for intervention, while disruption of the *Hif1α* gene with about 55% frameshift mutations was insufficient to suppress the development of CNV. And the long‐term therapeutic effect and potential side effects of this strategy need further exploring in a non‐human primate model or CTs for the further development of strategies for AMD. Besides, for the sake of clinical application, the selection of gene therapy targets and the time of medication intervention are likely to affect therapeutic effects and should be addressed in preclinical research exploration.

The precision and safety of gene editing therapies need to be improved to suppress VEGF expression and inhibit CNV development with minimal interference with the normal physiological function of RPE cells. The specificity of gene editor delivery strategies, such as the choice of local injection methods and AAV serotypes, expression specificity by specific gene expression promoters and the potential for off‐target effects and immunogenicity of Cas enzymes, are among the factors that need to be considered. For instance, the subretinal injection was used to target RPE cells for CNV inhibition, while the vitreous injection was preferable to prevent retinal neovascularization.^38^ To increase the transduction specificity of the gene editor, AAV vectors with a higher affinity for the target RPE cells were chosen, such as natural serotype AAV8, AAV9 or intentionally altered recombinant AAVs.^39^, ^40^, ^41^ Compared to LVs and adenoviruses, AAVs also have advantages such as relatively small size to easily diffuse in the retina, minimal integration into the genome, low immunogenicity and persistent expression in non‐dividing cells such as retinal neurons, making them one of the main vectors currently used for gene therapy applications.^42^, ^43^ On the other hand, to increase the effectiveness and accuracy of targeted editing in vivo, there is a need to screen small CRISPR‐Cas enzymes or variants and gRNAs with high precision that can be easily packaged by single AAVs, such as Nme_2_Cas9, *Staphylococcus* *aureus* Cas9, CjCas9 and so forth. Most Cas9 orthologs were detected with significant target editing effects in vivo 1 month after injection, as short as 2 weeks in the study of SaCas9 targeting the *CEP290* IVS26 site in mouse RPE cells.^44^ Our data show that Nme_2_Cas9 takes less time to produce significant editing effects in retinal cells than most of the reported Cas9 orthologs. The Nme_2_Cas9‐mediated in vivo gene editing achieved high editing efficiency without off‐target effects, which indicates that Nme_2_Cas9 is highly active, works rapidly and enables precise editing of target genes, potentially ameliorating safety issues resulting from off‐target activities.^18^, ^45^ It has been reported that the sustained expression of the gene editing system increases the off‐target nuclease genotoxicity and/or immune responses.^46^ Lipid nanoparticles (LNPs), a non‐viral nanoparticle delivery system widely used for RNA delivery, have shown great potential in message RNA (mRNA) vaccines.^47^ The safety of gene editing therapy for wet AMD will probably be further enhanced by using an LNP‐delivered Nme_2_Cas9 gene editing system that could specifically target retinal cells and transiently express gene editors to disrupt *Vegfa* gene expression. Current mRNA delivery applications, however, are primarily limited to the liver, and progress in developing selective organ‐targeted LNP was needed.^48^ It is encouraging that a recent study has identified some LNPs that can target RPE cells relatively specifically,^49^ but it remains to be seen whether they can efficiently deliver the gene editor in vivo and be used to treat ocular diseases.

## CONFLICT OF INTEREST STATEMENT

The authors declare no conflicts of interest.

## Supporting information

Supporting Information

## Data Availability

The NCBI Sequence Read Archive database (SRA; https://www.ncbi.nlm.nih.gov/sra) now has high‐throughput sequencing data that supported the conclusions of this study under accession numbers PRJNA915226 and PRJNA931838.
