# Supplementary material for: Nme2Cas9‐mediated therapeutic editing in inhibiting angiogenesis after wet age‐related macular degeneration onset
Source: Clin Transl Med. 2023 Aug 20;13(8):e1383. doi: 10.1002/ctm2.1383 (PMC10440058; doi:10.1002/ctm2.1383)
Supplement: Supplementary file 1 — Supporting Information [file CTM2-13-e1383-s001.pptx]

## Slide 1
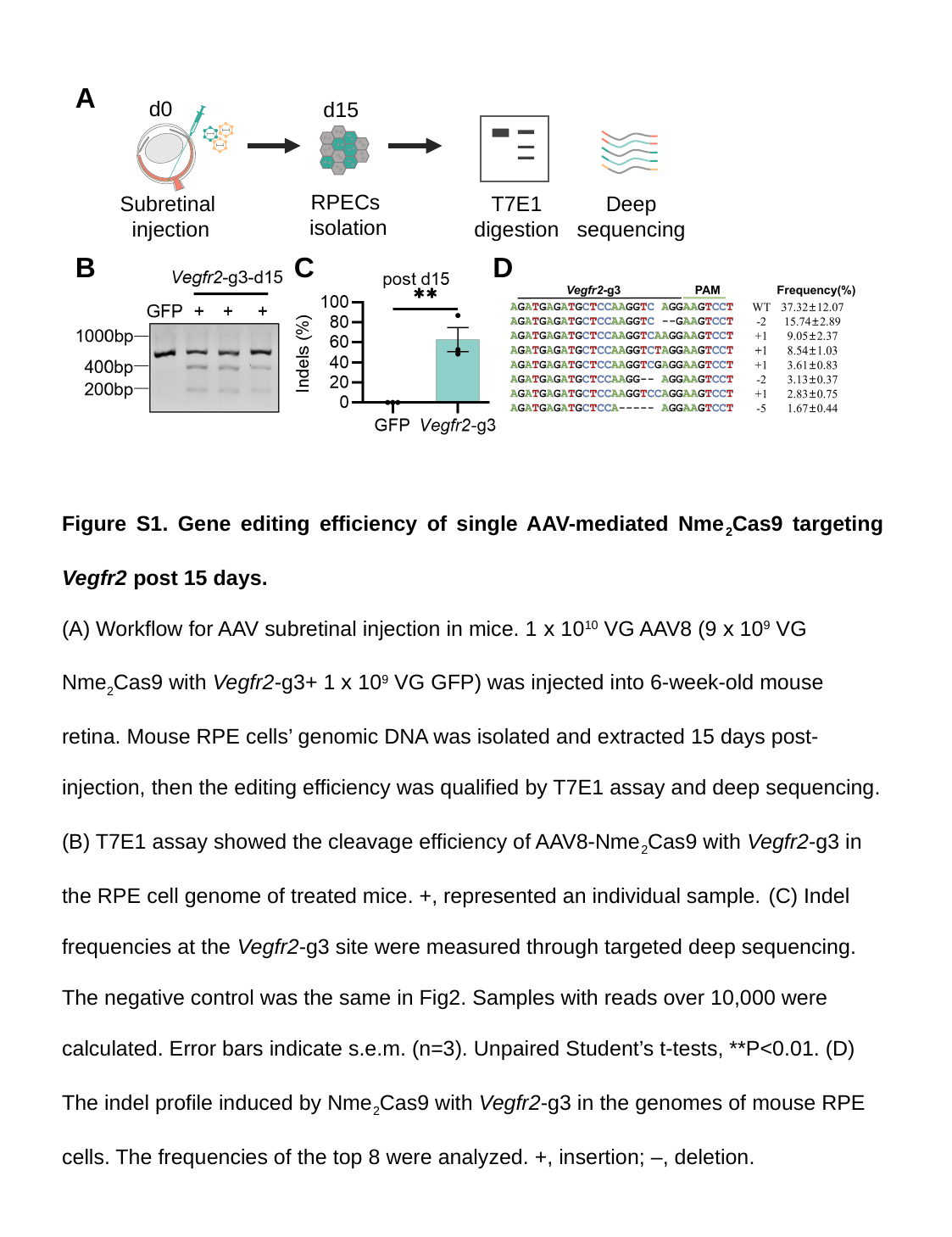

A
d0
d15
RPECs
isolation
Subretinal
injection
T7E1 digestion
Deep sequencing
C
D
B
Figure S1. Gene editing efficiency of single AAV-mediated Nme2Cas9 targeting Vegfr2 post 15 days.
(A) Workflow for AAV subretinal injection in mice. 1 x 10^10^ VG AAV8 (9 x 10^9^ VG Nme2Cas9 with Vegfr2-g3+ 1 x 10^9^ VG GFP) was injected into 6-week-old mouse retina. Mouse RPE cells’ genomic DNA was isolated and extracted 15 days post-injection, then the editing efficiency was qualified by T7E1 assay and deep sequencing. (B) T7E1 assay showed the cleavage efficiency of AAV8-Nme2Cas9 with Vegfr2-g3 in the RPE cell genome of treated mice. +, represented an individual sample. (C) Indel frequencies at the Vegfr2-g3 site were measured through targeted deep sequencing. The negative control was the same in Fig2. Samples with reads over 10,000 were calculated. Error bars indicate s.e.m. (n=3). Unpaired Student’s t-tests, **P<0.01. (D) The indel profile induced by Nme2Cas9 with Vegfr2-g3 in the genomes of mouse RPE cells. The frequencies of the top 8 were analyzed. +, insertion; –, deletion.

## Slide 2
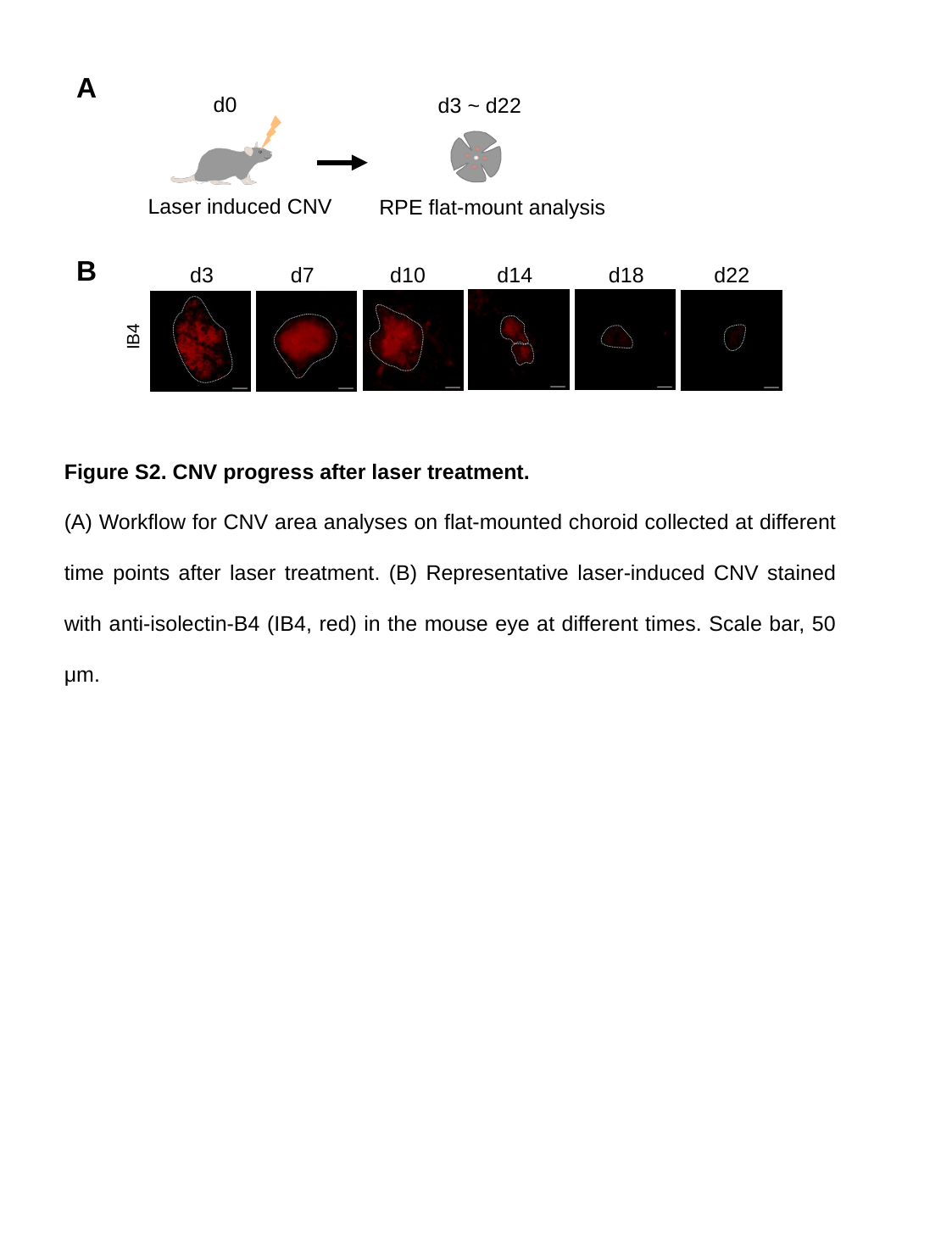

A
d0
d3 ~ d22
Laser induced CNV
RPE flat-mount analysis
B
d3
d7
d10
d14
d18
d22
IB4
Figure S2. CNV progress after laser treatment.
(A) Workflow for CNV area analyses on flat-mounted choroid collected at different time points after laser treatment. (B) Representative laser-induced CNV stained with anti-isolectin-B4 (IB4, red) in the mouse eye at different times. Scale bar, 50 μm.

## Slide 3
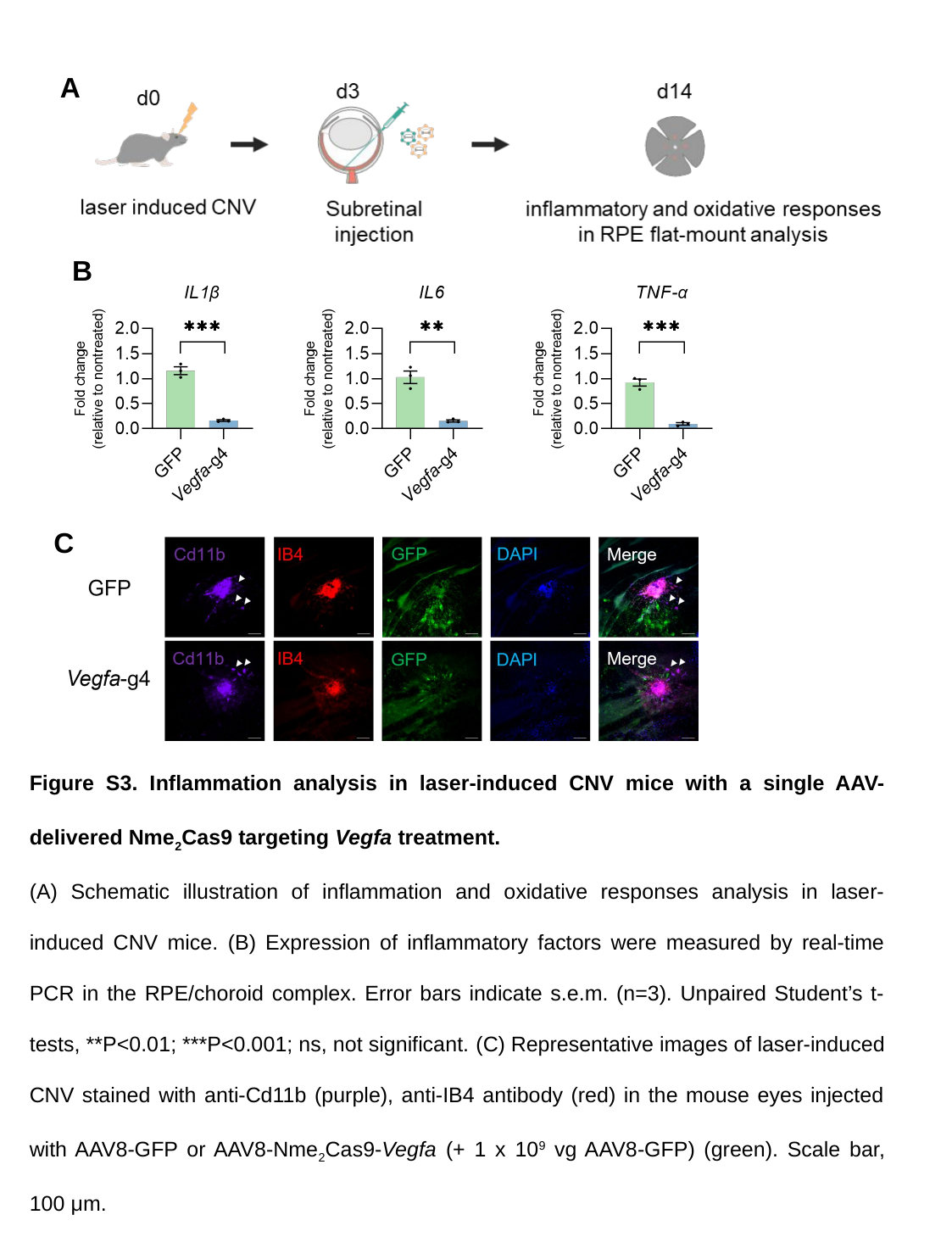

A
B
C
Figure S3. Inflammation analysis in laser-induced CNV mice with a single AAV-delivered Nme2Cas9 targeting Vegfa treatment.
(A) Schematic illustration of inflammation and oxidative responses analysis in laser-induced CNV mice. (B) Expression of inflammatory factors were measured by real-time PCR in the RPE/choroid complex. Error bars indicate s.e.m. (n=3). Unpaired Student’s t-tests, **P<0.01; ***P<0.001; ns, not significant. (C) Representative images of laser-induced CNV stained with anti-Cd11b (purple), anti-IB4 antibody (red) in the mouse eyes injected with AAV8-GFP or AAV8-Nme2Cas9-Vegfa (+ 1 x 10^9^ vg AAV8-GFP) (green). Scale bar, 100 μm.

## Slide 4
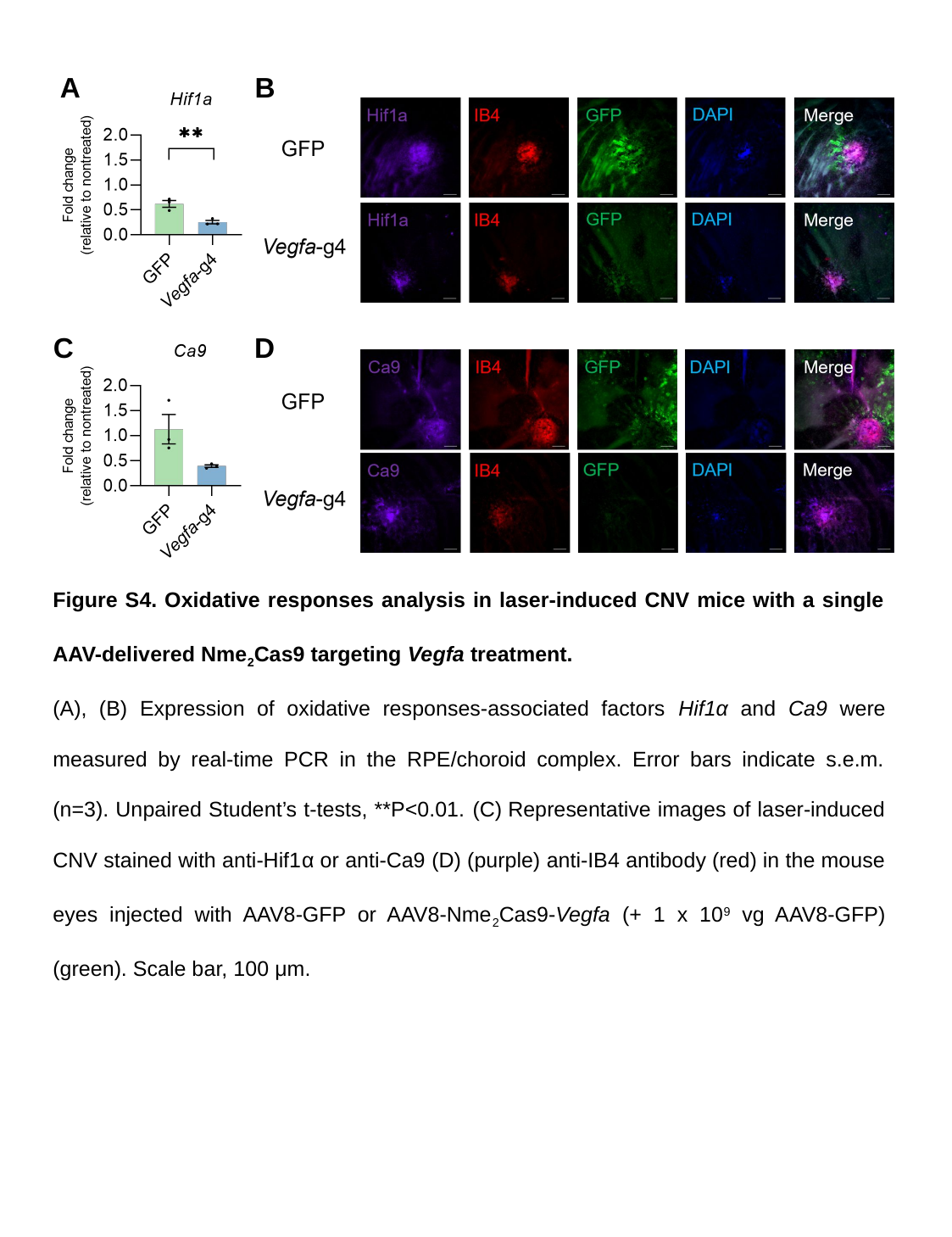

A
B
C
D
Figure S4. Oxidative responses analysis in laser-induced CNV mice with a single AAV-delivered Nme2Cas9 targeting Vegfa treatment.
(A), (B) Expression of oxidative responses-associated factors Hif1α and Ca9 were measured by real-time PCR in the RPE/choroid complex. Error bars indicate s.e.m. (n=3). Unpaired Student’s t-tests, **P<0.01. (C) Representative images of laser-induced CNV stained with anti-Hif1α or anti-Ca9 (D) (purple) anti-IB4 antibody (red) in the mouse eyes injected with AAV8-GFP or AAV8-Nme2Cas9-Vegfa (+ 1 x 10^9^ vg AAV8-GFP) (green). Scale bar, 100 μm.

## Slide 5
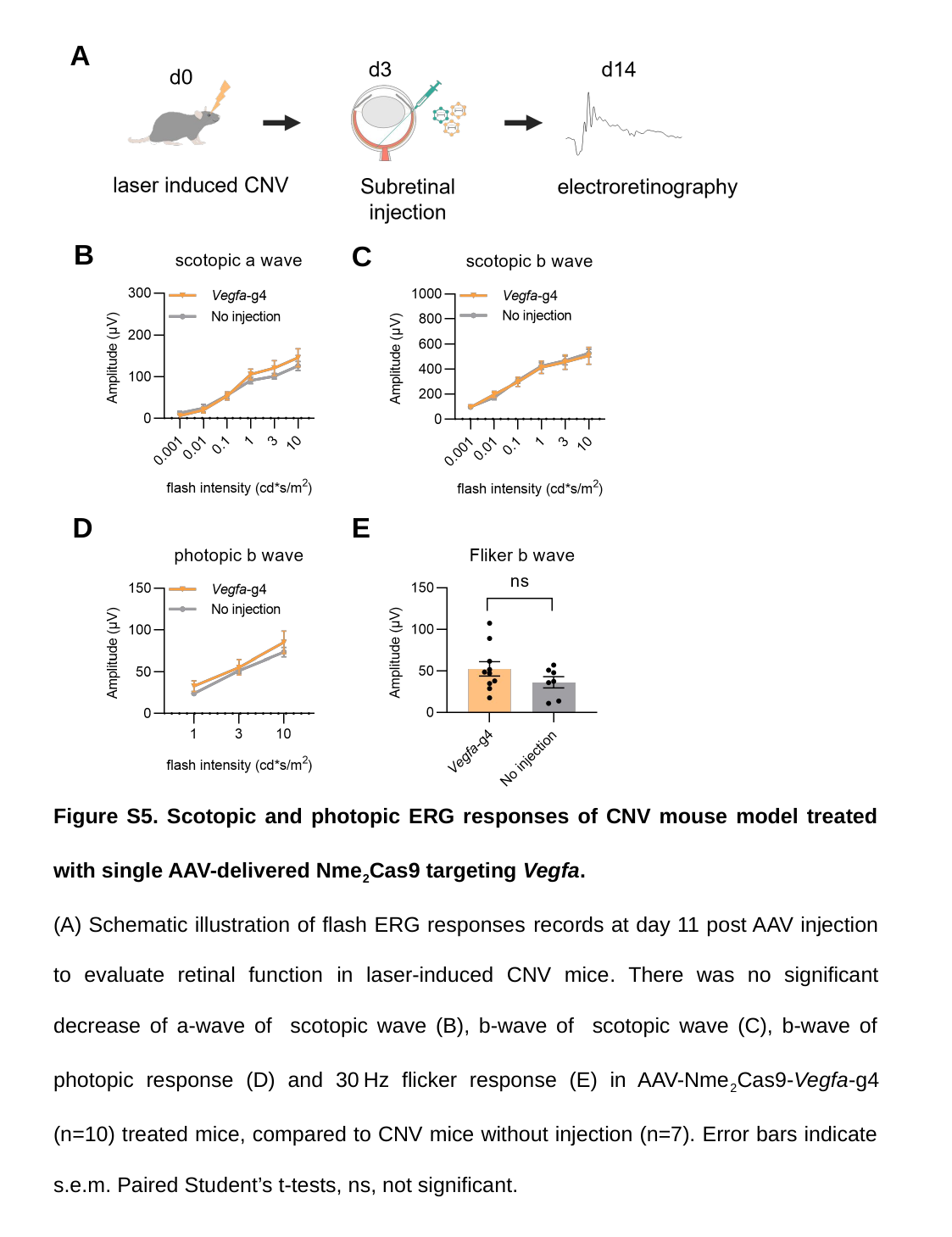

A
B
C
D
E
Figure S5. Scotopic and photopic ERG responses of CNV mouse model treated with single AAV-delivered Nme2Cas9 targeting Vegfa.
(A) Schematic illustration of flash ERG responses records at day 11 post AAV injection to evaluate retinal function in laser-induced CNV mice. There was no significant decrease of a-wave of scotopic wave (B), b-wave of scotopic wave (C), b-wave of photopic response (D) and 30 Hz flicker response (E) in AAV-Nme2Cas9-Vegfa-g4 (n=10) treated mice, compared to CNV mice without injection (n=7). Error bars indicate s.e.m. Paired Student’s t-tests, ns, not significant.

## Slide 6
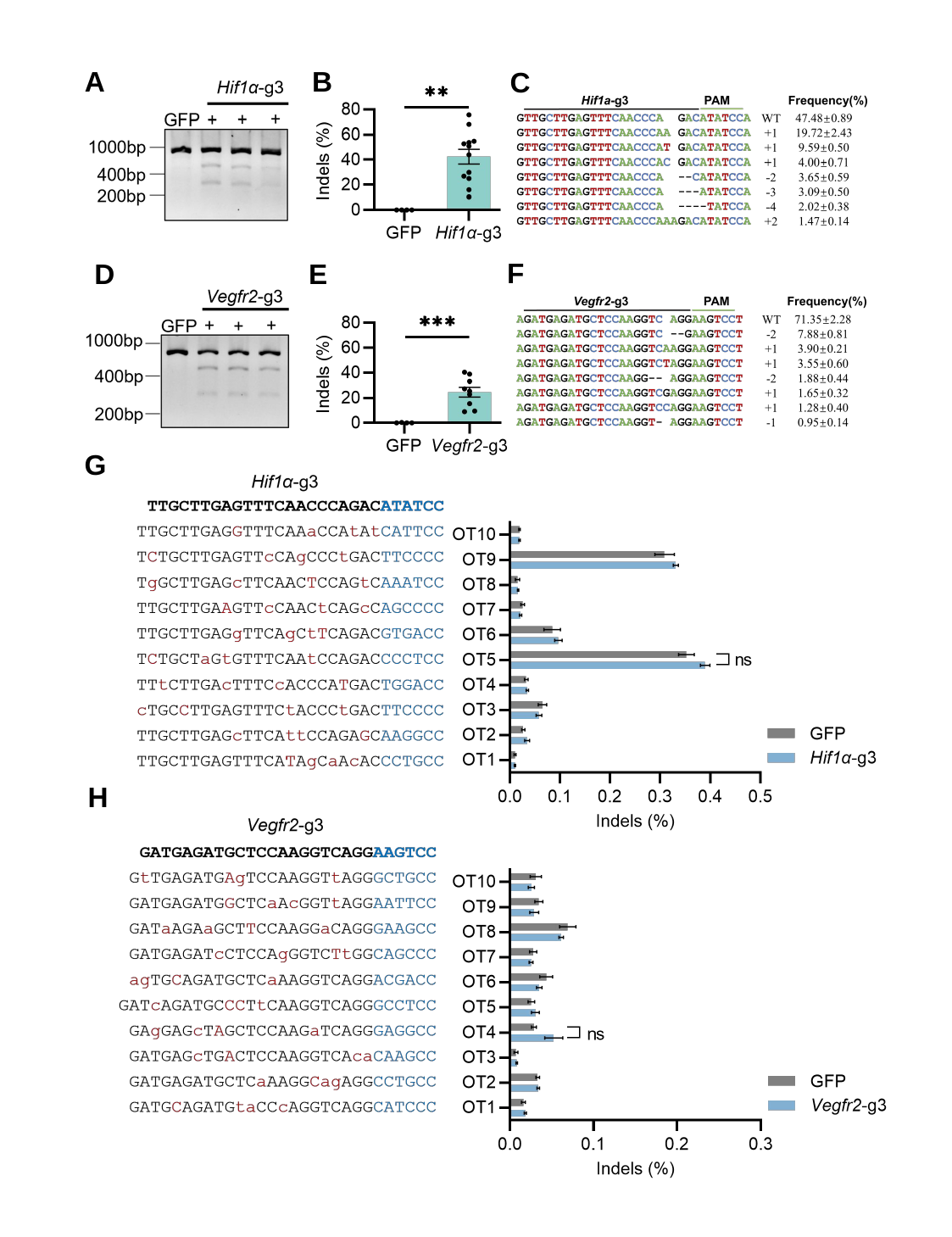

A
B
C
F
E
D
G
H

## Slide 7
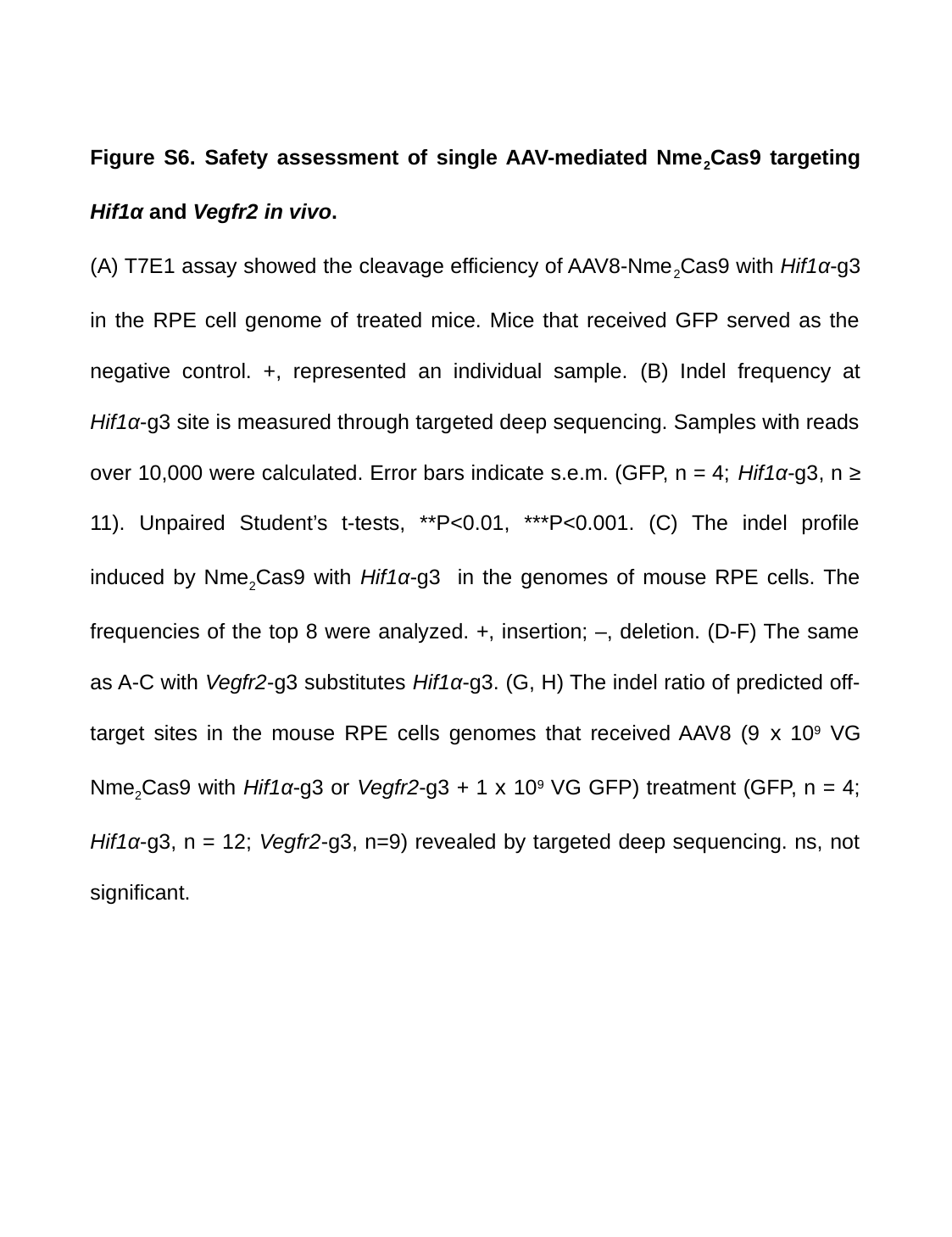

Figure S6. Safety assessment of single AAV-mediated Nme2Cas9 targeting Hif1α and Vegfr2 in vivo.
(A) T7E1 assay showed the cleavage efficiency of AAV8-Nme2Cas9 with Hif1α-g3 in the RPE cell genome of treated mice. Mice that received GFP served as the negative control. +, represented an individual sample. (B) Indel frequency at Hif1α-g3 site is measured through targeted deep sequencing. Samples with reads over 10,000 were calculated. Error bars indicate s.e.m. (GFP, n = 4; Hif1α-g3, n ≥ 11). Unpaired Student’s t-tests, **P<0.01, ***P<0.001. (C) The indel profile induced by Nme2Cas9 with Hif1α-g3 in the genomes of mouse RPE cells. The frequencies of the top 8 were analyzed. +, insertion; –, deletion. (D-F) The same as A-C with Vegfr2-g3 substitutes Hif1α-g3. (G, H) The indel ratio of predicted off-target sites in the mouse RPE cells genomes that received AAV8 (9 x 10^9^ VG Nme2Cas9 with Hif1α-g3 or Vegfr2-g3 + 1 x 10^9^ VG GFP) treatment (GFP, n = 4; Hif1α-g3, n = 12; Vegfr2-g3, n=9) revealed by targeted deep sequencing. ns, not significant.

## Slide 8
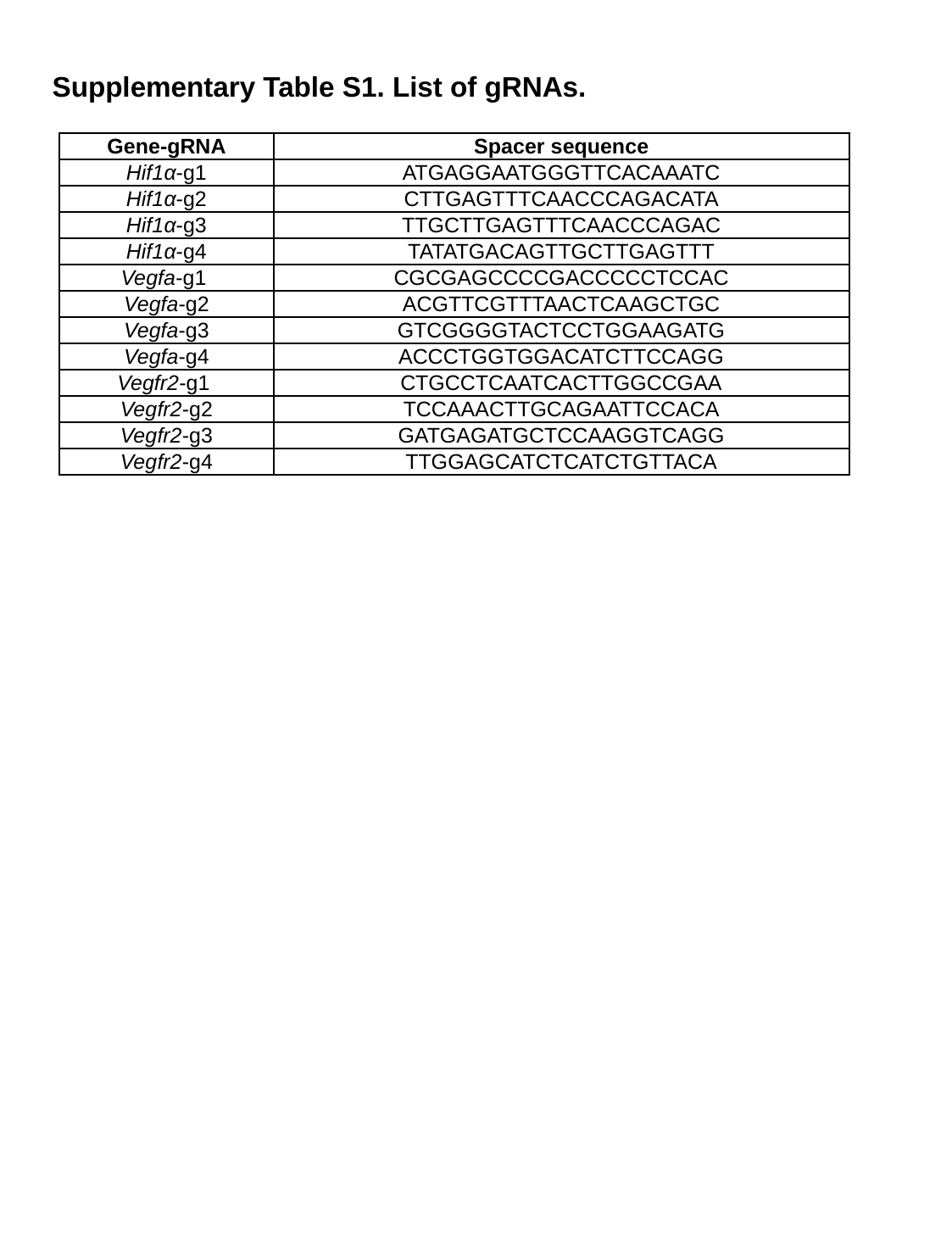

Supplementary Table S1. List of gRNAs.
| Gene-gRNA | Spacer sequence |
| --- | --- |
| Hif1α-g1 | ATGAGGAATGGGTTCACAAATC |
| Hif1α-g2 | CTTGAGTTTCAACCCAGACATA |
| Hif1α-g3 | TTGCTTGAGTTTCAACCCAGAC |
| Hif1α-g4 | TATATGACAGTTGCTTGAGTTT |
| Vegfa-g1 | CGCGAGCCCCGACCCCCTCCAC |
| Vegfa-g2 | ACGTTCGTTTAACTCAAGCTGC |
| Vegfa-g3 | GTCGGGGTACTCCTGGAAGATG |
| Vegfa-g4 | ACCCTGGTGGACATCTTCCAGG |
| Vegfr2-g1 | CTGCCTCAATCACTTGGCCGAA |
| Vegfr2-g2 | TCCAAACTTGCAGAATTCCACA |
| Vegfr2-g3 | GATGAGATGCTCCAAGGTCAGG |
| Vegfr2-g4 | TTGGAGCATCTCATCTGTTACA |

## Slide 9
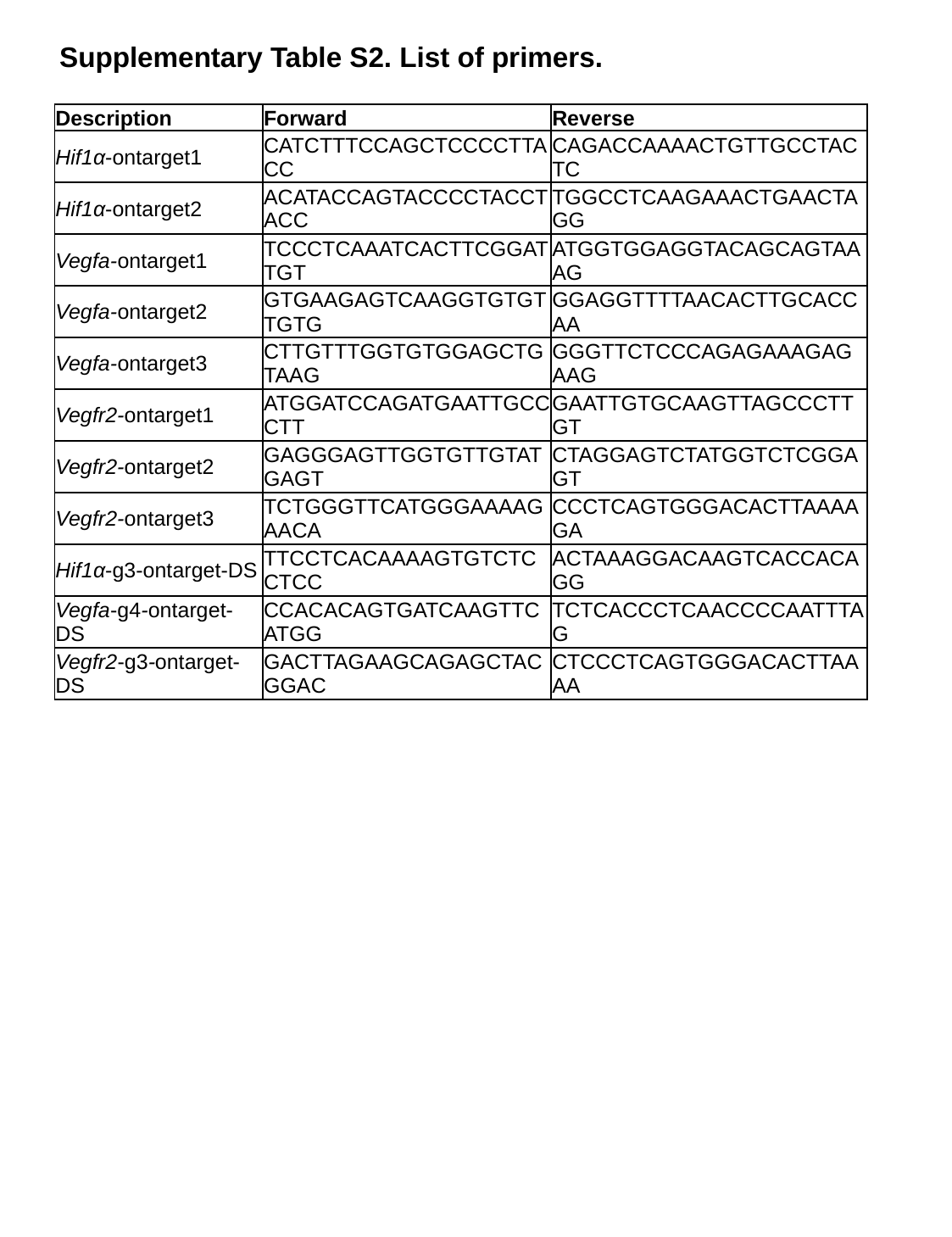

Supplementary Table S2. List of primers.
| Description | Forward | Reverse |
| --- | --- | --- |
| Hif1α-ontarget1 | CATCTTTCCAGCTCCCCTTACC | CAGACCAAAACTGTTGCCTACTC |
| Hif1α-ontarget2 | ACATACCAGTACCCCTACCTACC | TGGCCTCAAGAAACTGAACTAGG |
| Vegfa-ontarget1 | TCCCTCAAATCACTTCGGATTGT | ATGGTGGAGGTACAGCAGTAAAG |
| Vegfa-ontarget2 | GTGAAGAGTCAAGGTGTGTTGTG | GGAGGTTTTAACACTTGCACCAA |
| Vegfa-ontarget3 | CTTGTTTGGTGTGGAGCTGTAAG | GGGTTCTCCCAGAGAAAGAGAAG |
| Vegfr2-ontarget1 | ATGGATCCAGATGAATTGCCCTT | GAATTGTGCAAGTTAGCCCTTGT |
| Vegfr2-ontarget2 | GAGGGAGTTGGTGTTGTATGAGT | CTAGGAGTCTATGGTCTCGGAGT |
| Vegfr2-ontarget3 | TCTGGGTTCATGGGAAAAGAACA | CCCTCAGTGGGACACTTAAAAGA |
| Hif1α-g3-ontarget-DS | TTCCTCACAAAAGTGTCTCCTCC | ACTAAAGGACAAGTCACCACAGG |
| Vegfa-g4-ontarget-DS | CCACACAGTGATCAAGTTCATGG | TCTCACCCTCAACCCCAATTTAG |
| Vegfr2-g3-ontarget-DS | GACTTAGAAGCAGAGCTACGGAC | CTCCCTCAGTGGGACACTTAAAA |

## Slide 10
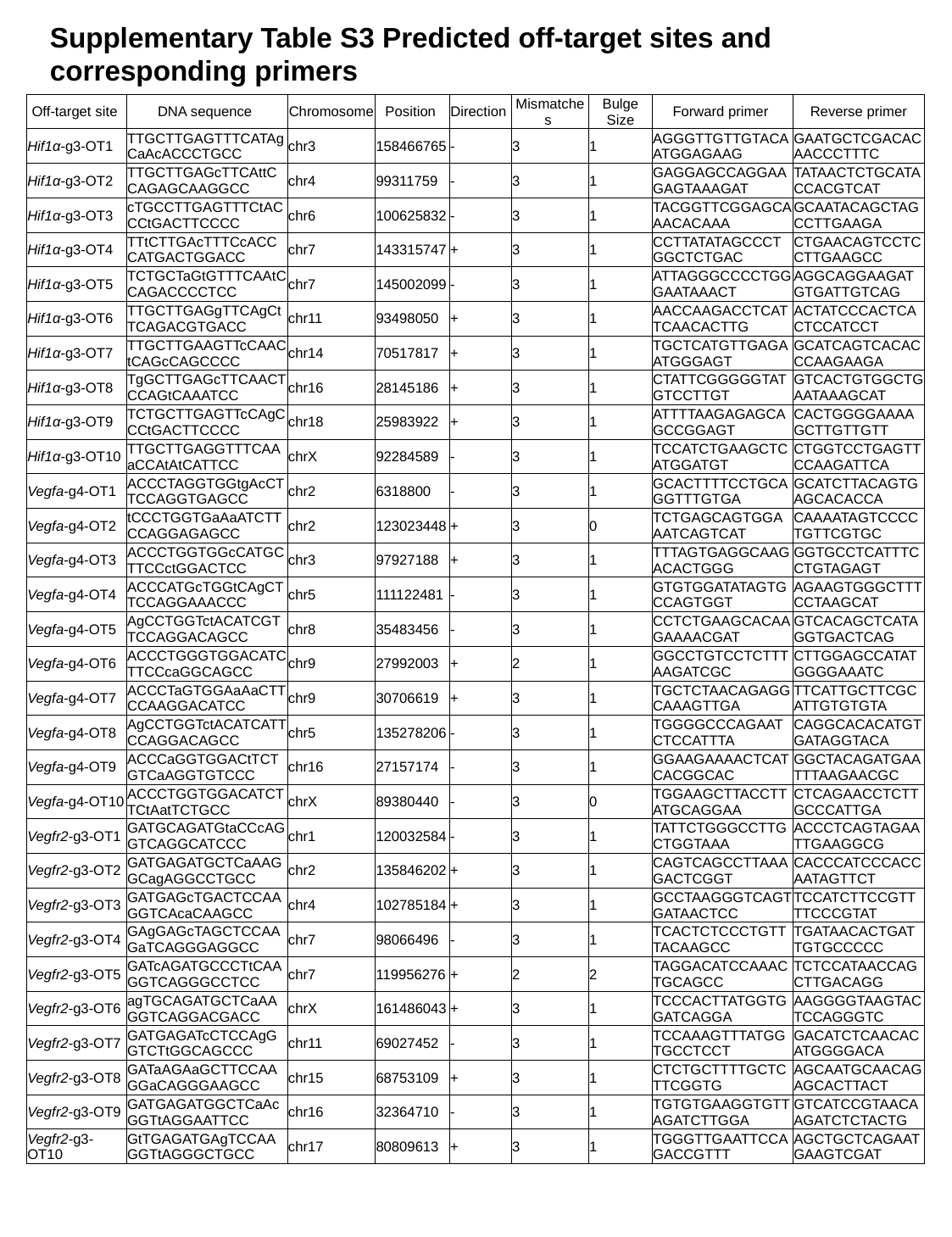

Supplementary Table S3 Predicted off-target sites and corresponding primers
| Off-target site | DNA sequence | Chromosome | Position | Direction | Mismatches | Bulge Size | Forward primer | Reverse primer |
| --- | --- | --- | --- | --- | --- | --- | --- | --- |
| Hif1α-g3-OT1 | TTGCTTGAGTTTCATAgCaAcACCCTGCC | chr3 | 158466765 | - | 3 | 1 | AGGGTTGTTGTACAATGGAGAAG | GAATGCTCGACACAACCCTTTC |
| Hif1α-g3-OT2 | TTGCTTGAGcTTCAttCCAGAGCAAGGCC | chr4 | 99311759 | - | 3 | 1 | GAGGAGCCAGGAAGAGTAAAGAT | TATAACTCTGCATACCACGTCAT |
| Hif1α-g3-OT3 | cTGCCTTGAGTTTCtACCCtGACTTCCCC | chr6 | 100625832 | - | 3 | 1 | TACGGTTCGGAGCAAACACAAA | GCAATACAGCTAGCCTTGAAGA |
| Hif1α-g3-OT4 | TTtCTTGAcTTTCcACCCATGACTGGACC | chr7 | 143315747 | + | 3 | 1 | CCTTATATAGCCCTGGCTCTGAC | CTGAACAGTCCTCCTTGAAGCC |
| Hif1α-g3-OT5 | TCTGCTaGtGTTTCAAtCCAGACCCCTCC | chr7 | 145002099 | - | 3 | 1 | ATTAGGGCCCCTGGGAATAAACT | AGGCAGGAAGATGTGATTGTCAG |
| Hif1α-g3-OT6 | TTGCTTGAGgTTCAgCtTCAGACGTGACC | chr11 | 93498050 | + | 3 | 1 | AACCAAGACCTCATTCAACACTTG | ACTATCCCACTCACTCCATCCT |
| Hif1α-g3-OT7 | TTGCTTGAAGTTcCAACtCAGcCAGCCCC | chr14 | 70517817 | + | 3 | 1 | TGCTCATGTTGAGAATGGGAGT | GCATCAGTCACACCCAAGAAGA |
| Hif1α-g3-OT8 | TgGCTTGAGcTTCAACTCCAGtCAAATCC | chr16 | 28145186 | + | 3 | 1 | CTATTCGGGGGTATGTCCTTGT | GTCACTGTGGCTGAATAAAGCAT |
| Hif1α-g3-OT9 | TCTGCTTGAGTTcCAgCCCtGACTTCCCC | chr18 | 25983922 | + | 3 | 1 | ATTTTAAGAGAGCAGCCGGAGT | CACTGGGGAAAAGCTTGTTGTT |
| Hif1α-g3-OT10 | TTGCTTGAGGTTTCAAaCCAtAtCATTCC | chrX | 92284589 | - | 3 | 1 | TCCATCTGAAGCTCATGGATGT | CTGGTCCTGAGTTCCAAGATTCA |
| Vegfa-g4-OT1 | ACCCTAGGTGGtgAcCTTCCAGGTGAGCC | chr2 | 6318800 | - | 3 | 1 | GCACTTTTCCTGCAGGTTTGTGA | GCATCTTACAGTGAGCACACCA |
| Vegfa-g4-OT2 | tCCCTGGTGaAaATCTTCCAGGAGAGCC | chr2 | 123023448 | + | 3 | 0 | TCTGAGCAGTGGAAATCAGTCAT | CAAAATAGTCCCCTGTTCGTGC |
| Vegfa-g4-OT3 | ACCCTGGTGGcCATGCTTCCctGGACTCC | chr3 | 97927188 | + | 3 | 1 | TTTAGTGAGGCAAGACACTGGG | GGTGCCTCATTTCCTGTAGAGT |
| Vegfa-g4-OT4 | ACCCATGcTGGtCAgCTTCCAGGAAACCC | chr5 | 111122481 | - | 3 | 1 | GTGTGGATATAGTGCCAGTGGT | AGAAGTGGGCTTTCCTAAGCAT |
| Vegfa-g4-OT5 | AgCCTGGTctACATCGTTCCAGGACAGCC | chr8 | 35483456 | - | 3 | 1 | CCTCTGAAGCACAAGAAAACGAT | GTCACAGCTCATAGGTGACTCAG |
| Vegfa-g4-OT6 | ACCCTGGGTGGACATCTTCCcaGGCAGCC | chr9 | 27992003 | + | 2 | 1 | GGCCTGTCCTCTTTAAGATCGC | CTTGGAGCCATATGGGGAAATC |
| Vegfa-g4-OT7 | ACCCTaGTGGAaAaCTTCCAAGGACATCC | chr9 | 30706619 | + | 3 | 1 | TGCTCTAACAGAGGCAAAGTTGA | TTCATTGCTTCGCATTGTGTGTA |
| Vegfa-g4-OT8 | AgCCTGGTctACATCATTCCAGGACAGCC | chr5 | 135278206 | - | 3 | 1 | TGGGGCCCAGAATCTCCATTTA | CAGGCACACATGTGATAGGTACA |
| Vegfa-g4-OT9 | ACCCaGGTGGACtTCTGTCaAGGTGTCCC | chr16 | 27157174 | - | 3 | 1 | GGAAGAAAACTCATCACGGCAC | GGCTACAGATGAATTTAAGAACGC |
| Vegfa-g4-OT10 | ACCCTGGTGGACATCTTCtAatTCTGCC | chrX | 89380440 | - | 3 | 0 | TGGAAGCTTACCTTATGCAGGAA | CTCAGAACCTCTTGCCCATTGA |
| Vegfr2-g3-OT1 | GATGCAGATGtaCCcAGGTCAGGCATCCC | chr1 | 120032584 | - | 3 | 1 | TATTCTGGGCCTTGCTGGTAAA | ACCCTCAGTAGAATTGAAGGCG |
| Vegfr2-g3-OT2 | GATGAGATGCTCaAAGGCagAGGCCTGCC | chr2 | 135846202 | + | 3 | 1 | CAGTCAGCCTTAAAGACTCGGT | CACCCATCCCACCAATAGTTCT |
| Vegfr2-g3-OT3 | GATGAGcTGACTCCAAGGTCAcaCAAGCC | chr4 | 102785184 | + | 3 | 1 | GCCTAAGGGTCAGTGATAACTCC | TCCATCTTCCGTTTTCCCGTAT |
| Vegfr2-g3-OT4 | GAgGAGcTAGCTCCAAGaTCAGGGAGGCC | chr7 | 98066496 | - | 3 | 1 | TCACTCTCCCTGTTTACAAGCC | TGATAACACTGATTGTGCCCCC |
| Vegfr2-g3-OT5 | GATcAGATGCCCTtCAAGGTCAGGGCCTCC | chr7 | 119956276 | + | 2 | 2 | TAGGACATCCAAACTGCAGCC | TCTCCATAACCAGCTTGACAGG |
| Vegfr2-g3-OT6 | agTGCAGATGCTCaAAGGTCAGGACGACC | chrX | 161486043 | + | 3 | 1 | TCCCACTTATGGTGGATCAGGA | AAGGGGTAAGTACTCCAGGGTC |
| Vegfr2-g3-OT7 | GATGAGATcCTCCAgGGTCTtGGCAGCCC | chr11 | 69027452 | - | 3 | 1 | TCCAAAGTTTATGGTGCCTCCT | GACATCTCAACACATGGGGACA |
| Vegfr2-g3-OT8 | GATaAGAaGCTTCCAAGGaCAGGGAAGCC | chr15 | 68753109 | + | 3 | 1 | CTCTGCTTTTGCTCTTCGGTG | AGCAATGCAACAGAGCACTTACT |
| Vegfr2-g3-OT9 | GATGAGATGGCTCaAcGGTtAGGAATTCC | chr16 | 32364710 | - | 3 | 1 | TGTGTGAAGGTGTTAGATCTTGGA | GTCATCCGTAACAAGATCTCTACTG |
| Vegfr2-g3-OT10 | GtTGAGATGAgTCCAAGGTtAGGGCTGCC | chr17 | 80809613 | + | 3 | 1 | TGGGTTGAATTCCAGACCGTTT | AGCTGCTCAGAATGAAGTCGAT |
